# Enhanced Gene Expression Rather than Natural Polymorphism in Coding Sequence of the *OsbZIP23* Determines Drought Tolerance and Yield Improvement in Rice Genotypes

**DOI:** 10.1371/journal.pone.0150763

**Published:** 2016-03-09

**Authors:** Avishek Dey, Milan Kumar Samanta, Srimonta Gayen, Soumitra K. Sen, Mrinal K. Maiti

**Affiliations:** 1 Adv. Lab. for Plant Genetic Engineering, Advanced Technology Development Center, Indian Institute of Technology Kharagpur, Kharagpur 721302, India; 2 Department of Biotechnology, Indian Institute of Technology Kharagpur, Kharagpur 721302, India; Louisiana State University Agricultural Center, UNITED STATES

## Abstract

Drought is one of the major limiting factors for productivity of crops including rice (*Oryza sativa* L.). Understanding the role of allelic variations of key regulatory genes involved in stress-tolerance is essential for developing an effective strategy to combat drought. The bZIP transcription factors play a crucial role in abiotic-stress adaptation in plants via abscisic acid (ABA) signaling pathway. The present study aimed to search for allelic polymorphism in the *OsbZIP23* gene across selected drought-tolerant and drought-sensitive rice genotypes, and to characterize the new allele through overexpression (OE) and gene-silencing (RNAi). Analyses of the coding DNA sequence (CDS) of the cloned *OsbZIP23* gene revealed single nucleotide polymorphism at four places and a 15-nucleotide deletion at one place. The single-copy *OsbZIP23* gene is expressed at relatively higher level in leaf tissues of drought-tolerant genotypes, and its abundance is more in reproductive stage. Cloning and sequence analyses of the *OsbZIP23*-promoter from drought-tolerant *O*. *rufipogon* and drought-sensitive IR20 cultivar showed variation in the number of stress-responsive *cis*-elements and a 35-nucleotide deletion at 5’-UTR in IR20. Analysis of the *GFP* reporter gene function revealed that the promoter activity of *O*. *rufipogon* is comparatively higher than that of IR20. The overexpression of any of the two polymorphic forms (1083 bp and 1068 bp CDS) of *OsbZIP23* improved drought tolerance and yield-related traits significantly by retaining higher content of cellular water, soluble sugar and proline; and exhibited decrease in membrane lipid peroxidation in comparison to RNAi lines and non-transgenic plants. The OE lines showed higher expression of target genes-*OsRab16B*, *OsRab21* and *OsLEA3-1* and increased ABA sensitivity; indicating that OsbZIP23 is a positive transcriptional-regulator of the ABA-signaling pathway. Taken together, the present study concludes that the enhanced gene expression rather than natural polymorphism in coding sequence of *OsbZIP23* is accountable for improved drought tolerance and yield performance in rice genotypes.

## Introduction

Plants are continuously exposed to wide ranges of environmental stresses such as drought, salinity, heat, low temperature and nutrient deficiency. The growth and developmental constraints due to such environmental stresses result in sub-optimal performance and reduced crop production. Drought is one such serious environmental stress which occurs when crop plants are subjected to insufficient soil moisture to meet their demands. Rice (*Oryza sativa* L.), which feeds more than half of the global population is adversely affected by soil moisture stress resulting in estimated 50% production loss worldwide [1]. Drought severely affects rice plants at morphological, physiological and molecular level. It is of vital importance to understand how plants respond to drought stress and what molecular mechanisms are being used to tackle this abiotic stress. Subsequently, the knowledge on the genetic factors or genes would help generating stress tolerant crop plants with better performance and improved yield. Allele mining is a promising way to identify naturally existing genetic variants of candidate genes, and thereby enabling direct access to key alleles conferring resistance to environmental stresses and to use such alleles for crop improvement programme.

The phytohormone abscisic acid (ABA), which is synthesized significantly under water deficit condition, plays a regulatory role in diverse physiological processes in plants coordinating a complex regulatory network that enables plant to cope with the decreased water availability [2–3]. The threshold level of ABA *in planta* is the central switch of drought tolerance, and controls various growth and developmental aspects such as cell division, seed germination, seedling growth, stomatal movement, synthesis of storage proteins and lipids and seed maturation. The ABA-dependent stress responses are regulated by the expression of stress-responsive genes [4–6]. The hierarchically upstream genetic regulators in this ABA stress-responsive pathway, mainly the transcription factors (TF) directly controls the expression of multiple other stress response related genes by binding to the *cis*-regulatory elements in their promoter region. The products from the later sets of genes are actually involved in biochemical and physiological functions to protect the plants against stresses. Thus, the TF genes are master regulators of stress responses. The basic leucine zipper (bZIP) TF family is one of the most diverse families in higher plants, whose members have different roles, particularly in stress responses and hormone signaling [7]. The members of the bZIP family contain the characteristic bZIP domain which is highly conserved and composed of a basic and a leucine zipper region [8]. They have been identified for their functions relating to the activation of hierarchically downstream gene expression by binding to the ACGT-motifs of ABA responsive elements (ABREs) in the promoter regions of these downstream genes [9]. Thus, these bZIP TFs were termed as ABRE-binding factors (ABFs) or ABRE-binding proteins (AREBs) [10].

In *Arabidopsis*, 75 identified bZIP TFs are subdivided into 10 groups. Group A bZIPs of *Arabidopsis* contains 13 members, many of them have been well studied and found to play central role in ABA-dependent stress signaling [11]. The group A subfamily III bZIPs in *Arabidopsis* were given special attentions (also known as ABF/AREB/ABI5) due to their biotechnological potentials. Among the seven members of these bZIP ABF genes, *AREB1*/*ABF2*, *AREB2*/*ABF4* and *ABF3* have been found to be expressed under abiotic stress condition, mainly in vegetative tissues [12–13]; and plants overexpressing these transcription factors showed enhanced drought tolerance [14–15]. The triple mutant of *areb1 areb2 abf3* showed highest level of decreased ABA sensitivity and reduced drought tolerance compared to single or double mutant, indicating that these TFs cooperatively regulate ABA stress signaling and require ABA for their complete activation [16]. The triple mutant of *areb1 arreb2 abf3* displayed remarkably impaired stress responsive gene expression leading to the identification of novel downstream factors, including late embryogenesis abundant (LEA) genes, group A type 2C phosphatase (PP2C) and numerous transcription factors [17]. In conclusion, the AREB/ABF genes are the major regulators of ABA-dependent gene expression in *Arabidopsis*.

Rice genome contains 89 putative bZIP TFs clustered into 11 groups based on their amino acid sequence similarity and DNA-binding specificity [18]. Several members have been studied for their functions related to stress responses including ABRE-binding factor *OSBZ8*, whose expression is strongly induced by ABA and positively correlated with salinity tolerance in rice [19]. The expression of *LIP19*, another member of rice bZIP TFs have been observed to be induced by low temperature, and it acts as a molecular switch in low temperature signaling pathway in rice [20]. Further, *RF2a* and *RF2b* bZIP TFs in rice have been found to be associated with biotic stress tolerance [21–22]. In comparison to comprehensive research on group A subfamily III bZIPs of *Arabidopsis*, relatively few orthologs in rice have been studied functionally for their roles in ABA-mediated stress responses, which include *TRAB1*, *OsABI5*, *OsbZIP23* and *OsbZIP46*. The *TRAB1* has been found to be activated by ABA-dependent phosphorylation and it interacts with *VP1* transcription factor [23]. The *OsABI5* has been suggested to be involved in ABA signaling, regulating fertility and stress responses [24]. The overexpression of *OsbZIP23* has conferred increased sensitivity to ABA and enhanced stress tolerance in rice [25]. Overexpression of intact *OsbZIP46* has exhibited no effect on drought tolerance; however a constitutively active form of *OsbZIP46* improved drought tolerance in rice upon overexpression [26]. Among the above-mentioned four members of group A subfamily III bZIPs of rice, the *OsbZIP23* is certainly the key player, which functions as a transcriptional activator and positively regulate the ABA-dependent stress responsive pathway, and hence increases the stress tolerance in rice [27].

Therefore, our present study aimed to search for natural allelic variations of the *OsbZIP23* gene across the selected drought-tolerant and drought-sensitive rice genotypes and few other members of the Poaceae family. After identifying the natural polymorphisms in the coding DNA sequence (CDS) and promoter regions of the *OsbZIP23* gene between drought-tolerant and drought-sensitive rice genotypes, we have functionally characterized the two promoter segments and two CDSs. The promoter activity has been investigated through *GFP* reporter gene expression analysis to find out the effect of natural polymorphism on *OsbZIP23* gene expression. On the other hand, the two allelic variants of *OsbZIP23* CDS have been studied through transgenic overexpression and RNAi-mediated endogenous gene silencing. The present study reveals that the *OsbZIP23*-mediated drought tolerance and yield improvement in rice genotypes depends on the enhanced gene expression due to increased promoter activity rather than the natural polymorphism in coding sequence.

## Materials and Methods

### Plant materials and growth conditions

Nine cultivated *indica* rice (*Oryza sativa L*.) genotypes, which include drought tolerant- Vandana, Manipuri, Nagina22 and drought sensitive- IR20, IR36, IR64. IR72, Swarna and HRC300 were chosen. Two wild progenitors of rice viz., *Oryza rufipogon*, *Oryza nivara* and four other representative members of the Poaceae family viz., *Zea mays* (maize), *Sorghum bicolor* (sorghum), *Pennisetum glaucum* (bajra) and a grass genotype *Brachypodium* was also included. All the selected genotypes were used for allelic polymorphism analysis. Rice genotypes were grown in the glass house at 25/28°C with a 16/8 h photoperiod and 70% relative humidity for all experimental works. For all phenotypic assays, seeds from the same harvest and same storage conditions were used.

### Drought stress treatment

To detect transcript level of *OsbZIP23* gene at vegetative and reproductive (grain filling) stages, leaf samples were collected from rice genotypes growing under three different conditions: (i) under normal watered condition that is referred as before stress (BS), (ii) under water stress (8 days) condition, which is referred as after stress (AS) and (iii) the rewatering (3 days) condition that is referred as after recovery (AR). Drought stress treatment in T_1_ transgenic lines and non-transgenic (NT) plants were performed under vegetative and early reproductive (panicle initiation) stages following the method reported earlier [28] with certain modification. For this, seeds of transgenic lines were germinated on MS agar medium containing 50mg/L hygromycin. The germinated transgenic and NT (germinated on MS agar only) seedlings were transferred into liquid ½ MS medium under 16h light/8h dark cycle at 25°C for one week. After one week, plants were transplanted into pots and grown for 20 days under standard growth conditions. Upon attaining vegetative and panicle initiation stages (about 35 days and 60 days of seeding, respectively) plants were subjected to drought stress by withholding water supply for a period of 8 days until a lethal effect of dehydration was observed on RNAi lines and NT plants. In each cases, rewatering was done for 3 days after the stress period and survival rate (%) of the transgenic lines and NT plants were determined as the number of visible green plants. For analysis of grain yield under drought stress in transgenic lines and NT plants, drought treatment at flowering stage was imposed in the PVC pipes as described previously [29]. Plants from each line were planted individually in the PVC pipe, and allowed to grow normally until they reach the flowering stage. Drought stress was initiated at flowering stage and after 10 days of water deprivation (when serious drought stress was visualized on RNAi lines and NT plants), plants were irrigated to allow recovery at the flowering and seed maturation stage. After the seed maturation, grain yield was calculated in transgenic lines and NT plants.

### Isolation of total cellular RNA and synthesis of 1^st^ strand cDNA

Total RNA from leaf tissues of rice plants was isolated using RNeasy Mini Kit (Qiagen) following the manufacturer’s instruction. First strand cDNA was synthesized from 2 μg of total RNA with gene-specific reverse primer using Transcriptor 1^st^ strand cDNA synthesis kit version 6.0 (Roche molecular biochemical) following manufacturer’s protocol.

### Isolation, cloning and sequence analysis of *OsbZIP23* coding DNA sequence

Isolation of the coding DNA sequence (CDS) of *OsbZIP23* gene was carried out by RT-PCR from leaf RNAs of drought-tolerant and drought-sensitive rice plants and non-rice (Poaceae family) plants mentioned in plant material section. The PCR was performed using 2 μl of the 1^st^ strand cDNA with gene-specific primer pairs bZ23F-bZ23R (S1 Table) by the following thermal profile: initial denaturation at 98°C for 2 min, followed by 30 cycles of 98°C/15 s, 60°C/15 s, 72°C/1 min and a final extension at 72°C for 8 min in a thermocycler (Applied Biosystems). The PCR products (~1083 bp size) were separately cloned in pUC18 plasmid. Nucleotide polymorphism of all the CDSs was analyzed after sequencing of positive clones. All the CDS-derived polypeptides were used for multiple amino acid sequence alignment through Jalview 2 [30].

### Real-time PCR analysis

Real-time PCR (qRT-PCR) for specific transcript was performed in Eppendorf Realplex^2^ Master Cycler using SYBR green based relative quantification method using 5 prime kits (Eppendorf). Three replicates were routinely used for each sample. Relative gene expression levels were determined as reported earlier [31]. Rice polyubiquitin1 gene (*OsUbi1*) was used as the internal control for normalization in each case. The primers used for real-time PCR are listed in S1 Table.

### Southern hybridization

The genomic DNA was isolated from leaf tissues of rice plants following the standard protocol [32]. For each sample, 15 μg of genomic DNA was digested with *Hind*III, run on 1% agarose gel overnight and transferred onto nylon membrane (Hybond-N^+^). A 739 bp DNA of *OsbZIP23* CDS (from *O*. *rufipogon*) was radiolabelled with P^32^-dCTP (3500 Ci/mmol) by random priming using rediprime II DNA labeling system (GE Healthcare, USA) following manufacturer’s instructions. Southern hybridization was performed [33] at 65°C in Church buffer (0.5 M phosphate buffer (pH-7.2), 1mM EDTA, 1% BSA and 7% SDS) using *OsbZIP23* gene probe. Similar procedure was used for Southern hybridization of transgenic rice lines developed in this study. The developed multi-sensitive X-ray film (Perkin Elmer) was scanned with a Cylone^®^ Plus phosphor system (Perkin Elmer).

### Isolation, cloning and *in silico* analysis of predicted *OsbZIP23* promoter

The predicted promoter sequence of ~1600 bp was PCR amplified from the genomic DNA isolated from leaves of *O*. *rufipogon* (drought-tolerant) and IR20 (drought-sensitive) rice genotypes using gene-specific primers bZ23PF-bZ23PR (S1 Table) by the following thermal profile: initial denaturation at 98°C for 2 min, followed by 30 cycles of 98°C/10 s, 55°C/15 s, 72°C/2 min and a final extension at 72°C for 8 min in a thermocycler (Applied Biosystems). The PCR products were then separately cloned in pUC18 plasmid, and positive clones were sequenced. The sequences of the cloned DNA fragments were analyzed for *cis*-acting elements and promoter core elements using PLACE [34] and Plant Promoter Database 3.0 [35] web tools.

### Generation of transgenic rice plants with the *OsbZIP23* promoter-*GFP* reporter gene construct and assay of GFP fluorescence by confocal microscopy

The *OsbZIP23* promoter-*GFP* reporter gene construct was prepared by fusing the putative *OsbZIP23* promoter DNA fragment of *O*. *rufipogon* (1612 bp) and IR20 (1578 bp) in *Hind*III and *Bam*HI restriction sites with *GFP* reporter gene followed by NOS transcription terminator in pCAMBIA1300 plasmid. The individual construct was introduced into drought-tolerant Vandana rice genotype through *Agrobacterium*-mediated transformation [36] and transgenic lines were confirmed by Southern hybridization using *GFP* reporter gene as hybridization probe. GFP fluorescence in leaves of transgenic rice lines was visualized with finely cross–sectioned leaf samples under the confocal microscope (Olympus FV1200). GFP was excited at 490 nm wavelength by 30 mW multi–Argon laser and the emission was at 520 nm. Images were taken at 40X magnification. The relative expression of *GFP* gene was monitored by qRT-PCR using specific primer set. The primers used are listed in S1 Table.

### Generation of transgenic rice plants with the *OsbZIP23* gene overexpression and gene silencing construct

For construction of the *OsbZIP23* overexpression plasmid, the full length CDS of allelic forms from *O*. *rufipogon* and *O*.*nivara* were put separately (in *Bam*HI and *Kpn*I restriction sites) under the rice polyubiquitin 1 (*OsUbi1*) promoter (*Hind*III and *Bam*HI restriction sites) and NOS transcription terminator (*Kpn*I and *Eco*RI restriction sites) in pCAMBIA1300 plasmid. Thus, two *OsbZIP2*3 overexpression (OE) constructs- OER for 1068 bp CDS of *O*. *rufipogon* and OEN for 1083 bp CDS of *O*. *nivara* were prepared. An RNAi-mediated gene silencing construct of *OsbZIP23* gene was also prepared. For this, a DNA fragment of 399 bp from 3' part of *OsbZIP23* CDS in sense (*Sal*I and *Bam*HI restriction sites) and antisense (*Bam*HI and *Kpn*I restriction sites) orientation separated by a linker DNA (*Bam*HI restriction site on both sides) was put under the *OsUbi1* promoter (*Hind*III and *Sal*I restriction site) and NOS transcription terminator (*Kpn*I and *Eco*RI restriction sites) in pCAMBIA1300 plasmid. The OEN, OER and RNAi constructs were introduced separately into drought-sensitive IR20 rice genotype through *Agrobacterium*-mediated transformation. The relative expression level of *OsbZIP23* gene in OEN, OER and RNAi transgenic rice lines was monitored by qRT-PCR using gene-specific primers. The primers used are listed in S1 Table.

### Immunoblotting for detection of OsbZIP23 protein in rice plants

The total protein was isolated from leaves of transgenic and NT rice plants and western blot analysis was carried out taking 40 μg of total protein following the previously reported method [37]. Affinity purified polyclonal antibody raised against OsbZIP23 protein in rabbit (catalog no- E580041-A-SE, EnoGene Biotech, USA) was used as primary antibody (1: 1,000 dilution) and monoclonal plant anti-actin antibody (Sigma, catalog no.-A0480) was used as loading control (1:500 dilution). Immuno-detection was carried out with the Lumi-LightPLUS western blotting kit (Roche Molecular Biochemicals), following manufacturer’s instruction.

### ABA sensitivity test during seed germination and seedling growth

For analysis of ABA sensitivity at seed germination stage, seeds of non-transgenic (NT) and transgenic rice plants were put on MS agar medium plates with different concentration of ABA (0, 1, 3and 6 μM), and allowed to germinate for 10 days. After 10 days, seed germination rates were calculated as percentage (%). Similarly for seedling stage, seeds of NT and transgenic plants were germinated on ½ MS liquid medium with different concentration of ABA (0, 1, 3 and 6 μM). The seedlings were grown for 14 days in a growth chamber (28 ± 2°C, 16/8 h photoperiod and 70% relative humidity) and shoot height and root length of seedlings were recorded.

### Measurement of water loss rate and relative water content in rice plants

Water loss rate (WLR) and relative water contents (RWC) were measured using leaves of transgenic and NT rice plants following reported method [38–39]. Two sets of experiment were performed for transgenic and NT plants and the results are consistent. The result from one set of experiment is presented. For WLR and RWC, each data point represents the average of three replicates.

### Measurement of proline and soluble sugar contents in leaf tissues of rice plants

Free proline content was determined using 50 mg of rice leaf tissue of transgenic and NT plants following the previously reported method [40]. Total soluble sugars in NT plants and transgenic lines were determined from 100 mg of rice tissues using anthrone reagent [41]. Two sets of experiment were performed for transgenic and NT plants and the results are consistent. The result from one set of experiment is presented. For proline and soluble sugars, each data point represents the average of three replicates.

### Measurement of malondialdehyde (MDA) contents and *in vivo* localization of reactive oxygen species (ROS) in leaf tissues of rice plants

The MDA content was determined using 100 mg of leaf tissue following the previously described method [42]. The absorbance (A) of the samples was measured at 450, 532 and 600 nm. The amount of MDA was calculated according to the formula: MDA content = 6.45× (*A*_532_–*A*_600_)–0.56×*A*_450_. Two sets of experiments were performed for transgenic and NT plants and the results are consistent. The result from one set of experiment is presented. For MDA content, each data point represents the average of three replicates. *In vivo* localization of reactive oxygen species (ROS) was done using the intact leaf samples following the method as reported earlier [43].

### Accession Numbers

Sequence data from this article can be accessed in the GenBank database with the following accession numbers: Rice polyubiquitin 1 (*OsUbi1*) gene (AF184279), *OsUbi1* promoter (AY785814), *OsbZIP23* promoter from *O*. *rufipogon* (KP779639), *OsbZIP23* promoter from IR20 (KP779640), *OsbZIP23* CDS from *O*. *rufipogon* (KP779637), *OsbZIP23* CDS from *O*. *nivara* (KP779638), *OsRab16B* (AF333275), *OsRab21* (Y00842) and *OsLEA3-1* (DQ789359).

## Results

### Analysis of allelic polymorphism in *OsbZIP23* CDS across selected rice genotypes and four other members of the Poaceae family differing in response to drought stress

**Fig 1:**
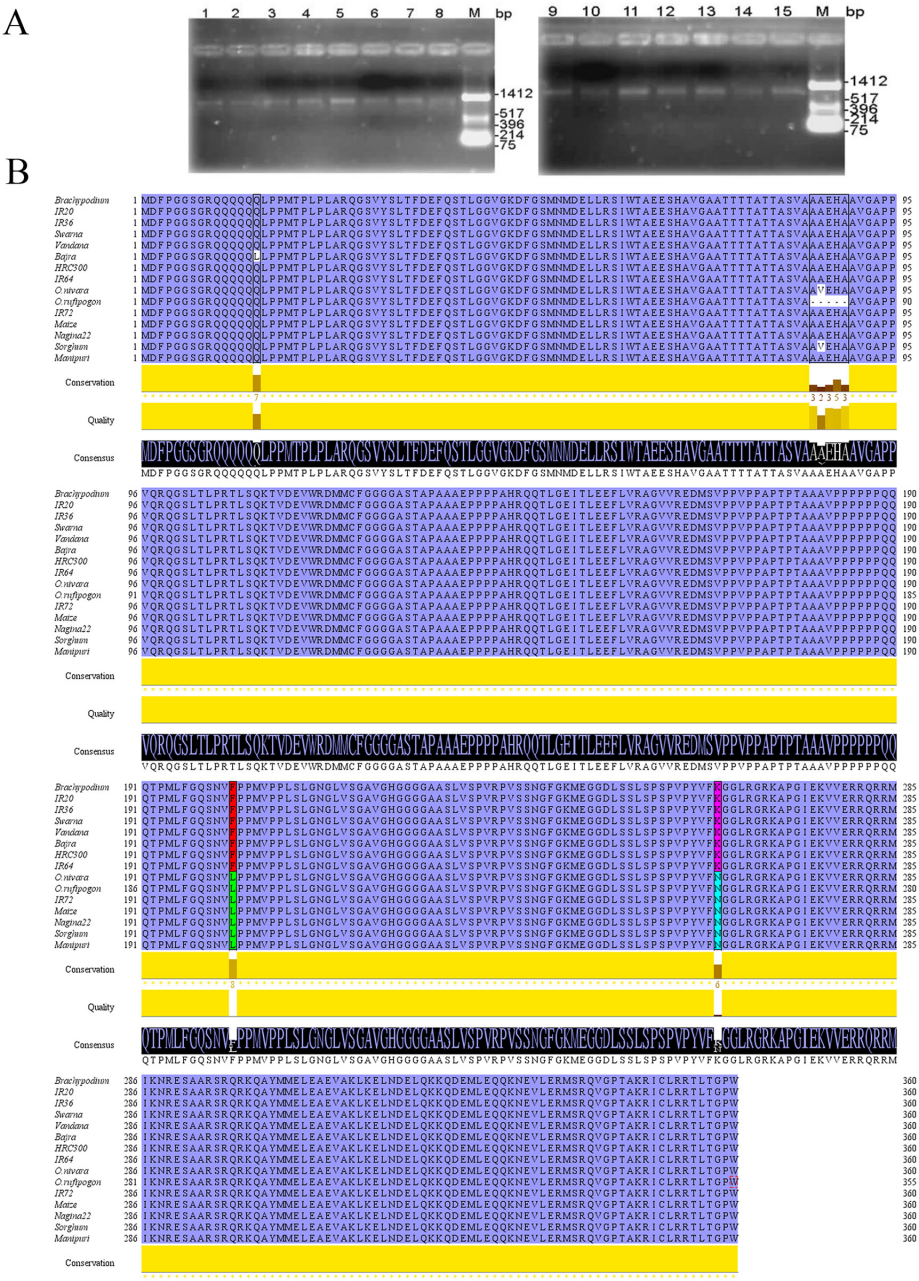
Analysis of natural polymorphism in the coding DNA sequence of *OsbZIP23* gene. **(A)** PCR amplification of *OsbZIP23* CDS from selected genotypes using gene-specific primers. Lanes 1–15 represents *O*. *rufipogon*, *O*. *nivara*, Vandana, Swarna, Nagina22, Manipuri, HRC300, IR20, IR36, IR64, IR72, sorghum, maize, *Brachypodium* and bajra respectively. Lane M- *Hin*fI digested pUC18 plasmid as a standard molecular weight marker. **(B)** Allelic polymorphism in the CDS of *OsbZIP23* gene across selected rice genotypes is represented by multiple sequence alignment in Jalview software.

For detection of allelic polymorphism in *OsbZIP23* gene, nine *indica* rice cultivars of drought-tolerant and drought-sensitive genotypes were selected along with two rice wild progenitors and four other representative members of the Poaceae family. The full length coding DNA sequence (CDS) of *OsbZIP23* was amplified from leaf RNA samples of selected genotypes through RT-PCR using the gene-specific primers. The amplicons were found to have an expected size of ~1083 bp (Fig 1A). The amplicons from each genotype was cloned in pUC18 plasmid and positive clones were sequenced. Multiple sequence alignment of the CDS and the derived polypeptide of *OsbZIP23* across the 11 rice genotypes and four other members of the Poaceae family revealed the presence of natural polymorphism (Fig 1B). Four non-synonymous single nucleotide polymorphisms (SNPs) and one 15-nucleotide deletion were detected. First SNP at position 606^th^ (C to A) from the start codon led to 202^nd^ amino acid alteration from Phenylalanine (F) to Leucine (L), and the 2^nd^ SNP at position 789^th^ (A to T) from the start codon led to 263^rd^ amino acid alteration from Lysine (K) to Asparagine (N). Phenylalanine and Lysine were present at 202^nd^ and 263^rd^ amino acid positions, respectively in IR20, IR36, IR64, Swarna, Vandana, HRC300, bajra and *Brachypodium*; whereas Leucine and Asparagine were present at the corresponding positions in IR72, Nagina22, Manipuri, *O*. *rufipogon*, *O*. *nivara*, maize and sorghum. The 3^rd^ SNP was observed in the *OsbZIP23* CDS of bajra, where nucleotide change (A to T) at position 44^th^ from the start codon altered the 15^th^ amino acid from Glutamine (Q) to Leucine (L). The 4^th^ SNP was observed in the *OsbZIP23* CDS of *O*. *nivara* and sorghum, where nucleotide change (C to T) at the position 257^th^ from the start codon led to an altered amino acid at 86^th^ position from Alanine (A) to Valine (V). Interestingly, a major 15-nucleotide deletion resulting in elimination of five amino acids from position 85^th^ to 89^th^ in the N-terminal region of OsbZIP23 was observed in wild progenitor *O*. *rufipogon* (Fig 1B). Although the 5-amino acid deletion sequence of *O*. *rufipogon* is AAEHA in almost all the genotypes tested, but this sequence is AVEHA in *O*. *nivara* and sorghum (Fig 1B).

### Analyses of relative expression and copy number of *OsbZIP23* gene in the selected rice genotypes

**Fig 2:**
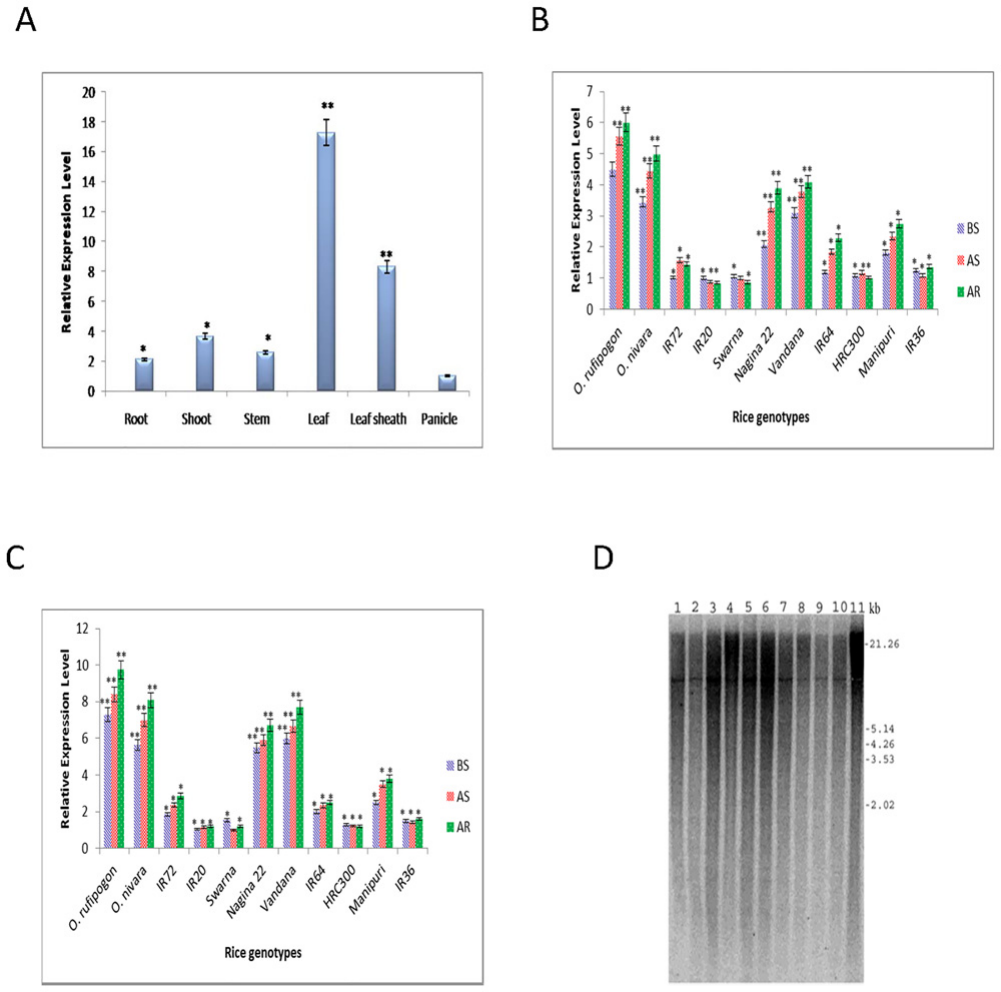
Analysis of the transcript expression level and copy number of the endogenous *OsbZIP23* gene. **(A)** Relative expression level of *OsbZIP23* gene in different tissues of an upland *indica* rice genotype, Vandana. Real time PCR analysis of *OsbZIP23* transcript in vegetative **(B)** and reproductive (grain filling) stage **(C)**, in 11 selected rice genotypes grown under before stress (BS), after stress (AS) and after recovery (AR) condition as described in method section. Rice polyubiquitin1 (*OsUbi1*) gene was taken as internal reference. Data bars represent the mean ±SD of triplicate measurement. Statistical analysis of Student’s t test indicated significant differences (* P<0.05, **P<0.01). **(D)** Southern blot showing the single copy of endogenous *OsbZIP23* gene in 11 selected rice genotypes. Lanes 1–11 represent *O*. *rufipogon*, *O*. *nivara*, Vandana, IR20, Swarna, IR36, IR64, IR72, Nagina22, Manipuri, HRC300 rice genotypes, respectively.

To investigate the *OsbZIP23* gene expression patterns in different tissues of the rice plant, we performed qRT-PCR analyses using the total RNA isolated from various tissues of Vandana, a cultivated drought-tolerant *indica* rice genotype. The *OsbZIP23* transcript was found to be present in all tested tissues, including root, shoot, stem, leaf, leaf sheath and panicle, with highest transcript expression levels in leaf and leaf sheath than in other tissues (Fig 2A). The allelic polymorphism in the coding region of *OsbZIP23* gene impelled us to check the relative expression level of this gene by qRT-PCR in the selected rice genotypes, which include nine cultivated *indica* rice genotypes viz., Vandana, Nagina22, Manipuri, Swarna, HRC300, IR20, IR36, IR64, IR72 and two wild progenitors of rice *O*. *rufipogon* and *O*. *nivara*. For this analysis, rice genotypes under the vegetative and reproductive (grain filling) stages were selected. Total RNA was isolated from leaves of all the rice genotypes grown under three different conditions: (i) before stress (BS), (ii) after stress (AS) and (iii) after recovery (AR). The qRT-PCR analyses showed that the relative expression level of *OsbZIP23* transcript increased in AS and AR samples of drought-tolerant genotypes *O*. *rufipogon*, *O*. *nivara*, Nagina22, Vandana and Manipuri in both vegetative (Fig 2B) and reproductive stages (Fig 2C). However, the relative expression levels of *OsbZIP23* transcript in AS and AR samples of drought-sensitive genotypes IR20, IR36, IR64, IR72, HRC300 and Swarna were not much comparable to that of BS samples in both vegetative (Fig 2B) and reproductive stages (Fig 2C). It was also observed that the abundance of *OsbZIP23* transcript in all the genotypes were comparatively higher in reproductive stage (Fig 2C) than in the vegetative stage (Fig 2B). The differences in the relative expression level of *OsbZIP23* transcript in the selected rice genotypes grown under the same conditions, led us to check the copy number of *OsbZIP23* gene in the genome of all the selected rice genotypes through Southern blot hybridization using *OsbZIP23* gene-specific probe. Southern blot analysis revealed a single band signal with *Hind*III digested genomic DNA samples in all the selected rice genotypes (Fig 2D), indicating the presence of only one copy of this gene in all the genotypes tested.

### *In silico* analysis of the predicted promoter sequence of *OsbZIP23* gene from drought tolerant and drought sensitive rice genotypes

**Fig 3:**
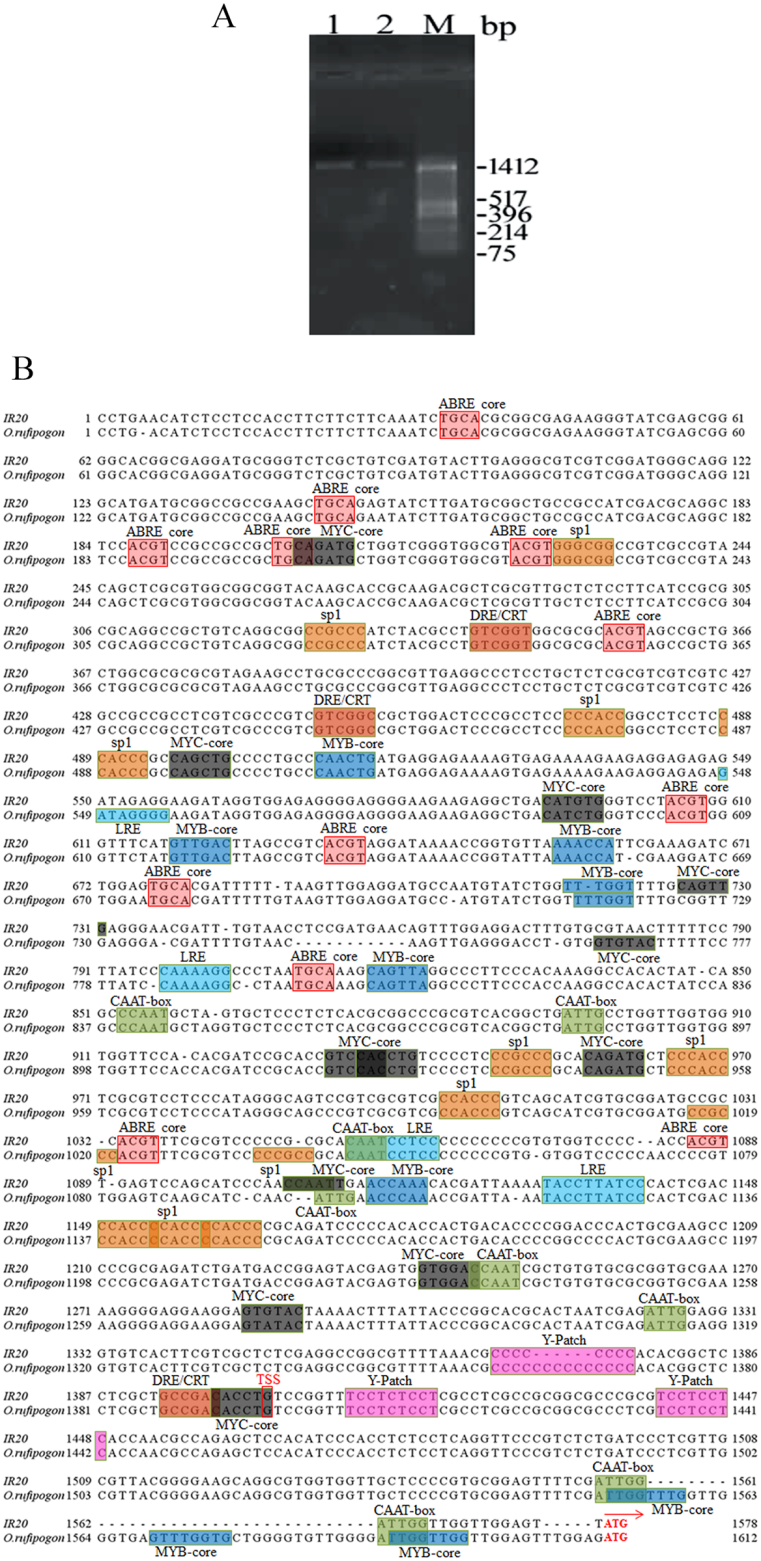
Cloning and *in silico* analysis of *OsbZIP23* promoter sequences. **(A)** PCR amplification of the *OsbZIP23* gene promoter region from two selected rice genotypes using gene-specific primers. Lanes 1–2 represent *O*. *rufipogon* and IR20, respectively. Lane M- *Hin*fI digested pUC18 plasmid as a standard molecular weight marker. **(B)** Alignment of nucleotide sequences and distribution of major *cis*-regulatory elements in the *OsbZIP23* promoter of *O*. *rufipogon* and IR20.

The wide variation in the relative expression level of *OsbZIP23* transcript between the drought-tolerant wild progenitor *O*. *rufipogon* and drought-sensitive genotype IR20 prompted us to analyze the promoter region of *OsbZIP23* gene from these two rice genotypes. For this, ~1.6 kb DNA fragment upstream of the start codon was PCR amplified from the two genomic DNA samples using the gene-specific primers (Fig 3A), cloned and sequenced. Alignment of the predicted *OsbZIP23* promoter sequences from *O*. *rufipogon* and IR20 showed a major deletion of 35 nucleotide (17 nucleotide upstream of the start codon) in the 5'-UTR segment of IR20 genotype in comparison to *O*. *rufipogon* (Fig 3B). Searching for the regulatory motifs in the promoter sequence using the PLACE database predicted several putative stress response-related *cis*-elements, which vary in number within *O*. *rufipogon* and IR20 genotypes. ABA-responsive element (ABRE) core has 11 hits in *O*. *rufipogon* and 12 hits in IR20; dehydration responsive element (DRE) has 3 hits in both the genotypes. Among other *cis*-elements MYC-core is present 10 times in *O*. *rufipogon* and 11 times in IR20, whereas MYB-core occurs 9 times in *O*. *rufipogon* and 7 times in IR20. Interestingly, the 35-nucleotide difference causes two extra MYC-cores in *O*. *rufipogon* (Fig 3B). Apart from these stress responsive *cis*-elements, some light responsive elements (LRE) were also found to be present in both the promoter sequences. Analysis revealed that the predicted transcription start site (TSS) is present at 216 bp upstream from the ATG codon in case of *O*. *rufipogon*, compared to 181 bp upstream in case of IR20. The *OsbZIP23* promoter was predicted to be TATA-less, but the core promoter elements like CCAAT-box and Y-patches were found to be present around TSS in both the sequences.

### The *OsbZIP23* gene promoter from the drought tolerant genotype drives higher level of the reporter gene expression than that of drought sensitive one

**Fig 4:**
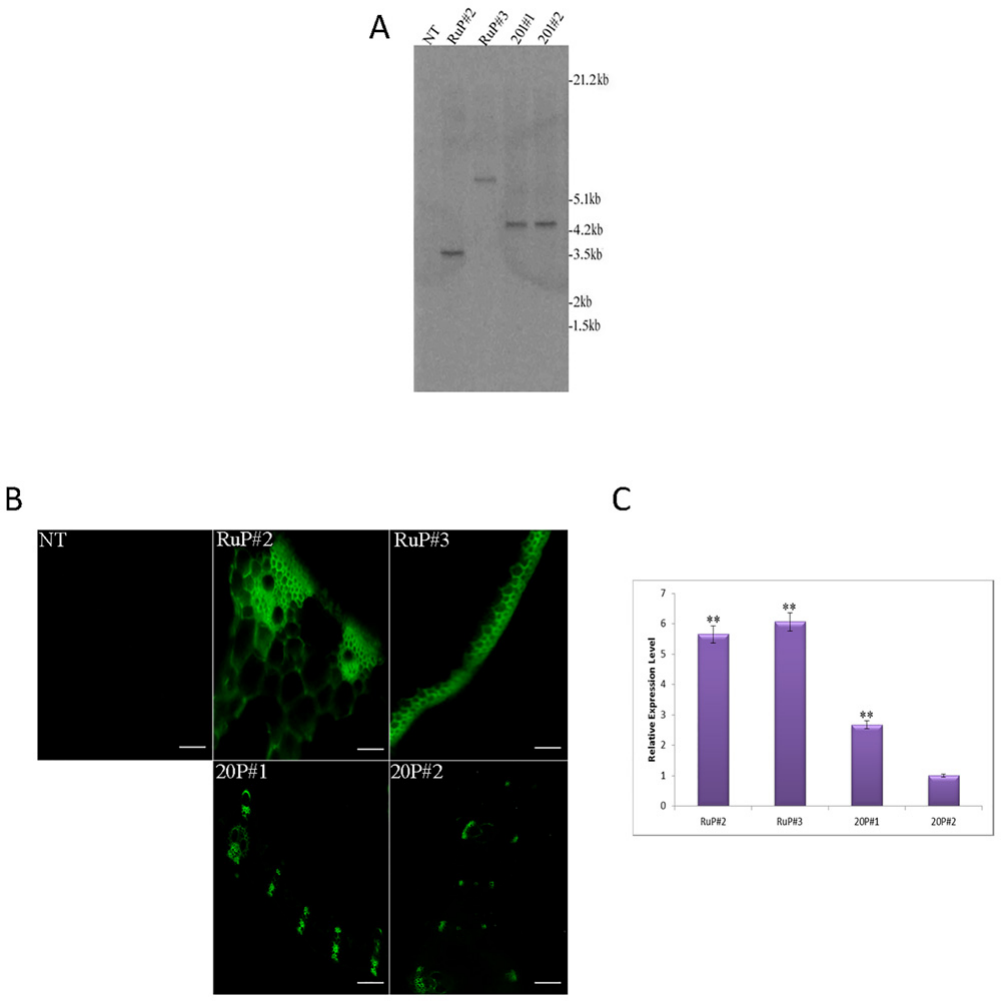
Southern blot and GFP fluorescence analyses of transgenic and NT plants for studying the promoter activity of *OsbZIP23* gene. **(A)** Southern blot analysis of rice T_0_ transformants of RuP and 20P lines developed with the *O*. *rufipogon* promoter-GFP construct (S1 Fig) and *O*. *sativa* IR20 promoter-GFP construct (S2 Fig), respectively. The genomic DNA of plant sample was digested with *Hind*III, and the CDS of GFP gene was used as hybridization probe. Lane NT- non-transgenic, Lane M- molecular weight marker. (**B**) GFP expression in finely cross-sectioned leaves of drought stressed single integration transgenic rice lines under the confocal microscope (Olympus FV1200). Scale bar 50 μm. (**C**) Real time PCR analysis showing relative expression level of *GFP* in leaf samples of RuP and 20P lines under drought stress condition, where rice polyubiquitin1 (*OsUbi1*) gene was taken as internal reference. Data bars represent the mean ±SD of triplicate measurement. Statistical analysis of Student’s t test indicated significant differences (**P<0.01).

To examine the differences in *OsbZIP23* promoter activity, if any, between *O*. *rufipogon* and IR20 rice genotypes, two *GFP* reporter gene constructs were prepared (S1 and S2 Figs) using the *OsbZIP23* promoter fragment isolated from *O*. *rufipogon* and IR20, and introduced into drought-tolerant Vandana rice genotype through *Agrobacterium*-mediated transformation. The tissue culture regenerated plantlets were screened in hygromycin and subjected to Southern blot analysis, which revealed single integration transgenic lines (Fig 4A). The transformed lines developed with the *OsbZIP23* promoter-*GFP*-NOS construct in case of *O*. *rufipogon* and IR20 were designated as RuP and 20P, respectively. The T_0_ seeds from the respective lines were germinated in hygromycin containing media and Southern blot analysis was performed, which showed similar transgene integration pattern in T_1_ plants (data not shown) as it was observed in T_0_ plants (Fig 4A). The Southern positive T_1_ plants were used for further analyses. To check the activity of both the promoters, the GFP fluorescence was observed in cross-sectioned leaf samples collected under drought stress condition from the transgenic RuP and 20P lines using confocal microscopy. It was found that the GFP fluorescence in RuP#2 and RuP#3 lines were comparatively higher than the 20P#1 and 20P#2 lines, whereas the non-transgenic (NT) plants showed no GFP fluorescence (Fig 4B). Further to quantitate the relative expression level of *GFP* in all the four lines, qRT-PCR analysis was carried out taking 20P#2 as calibrator (the least GFP fluorescence was observed in 20P#2 line). The qRT-PCR analysis revealed that the relative expression level of *GFP* in RuP#2 and RuP#3 lines were significantly (P>0.01) greater than the 20P#1 line (Fig 4C). These results indicated that the *OsbZIP23* promoter isolated from the drought-tolerant *O*. *rufipogon* genotype exhibits higher functional activity than the drought-sensitive IR20 genotype.

### Generation of *OsbZIP23* overexpression and silencing transgenic rice lines

**Fig 5:**
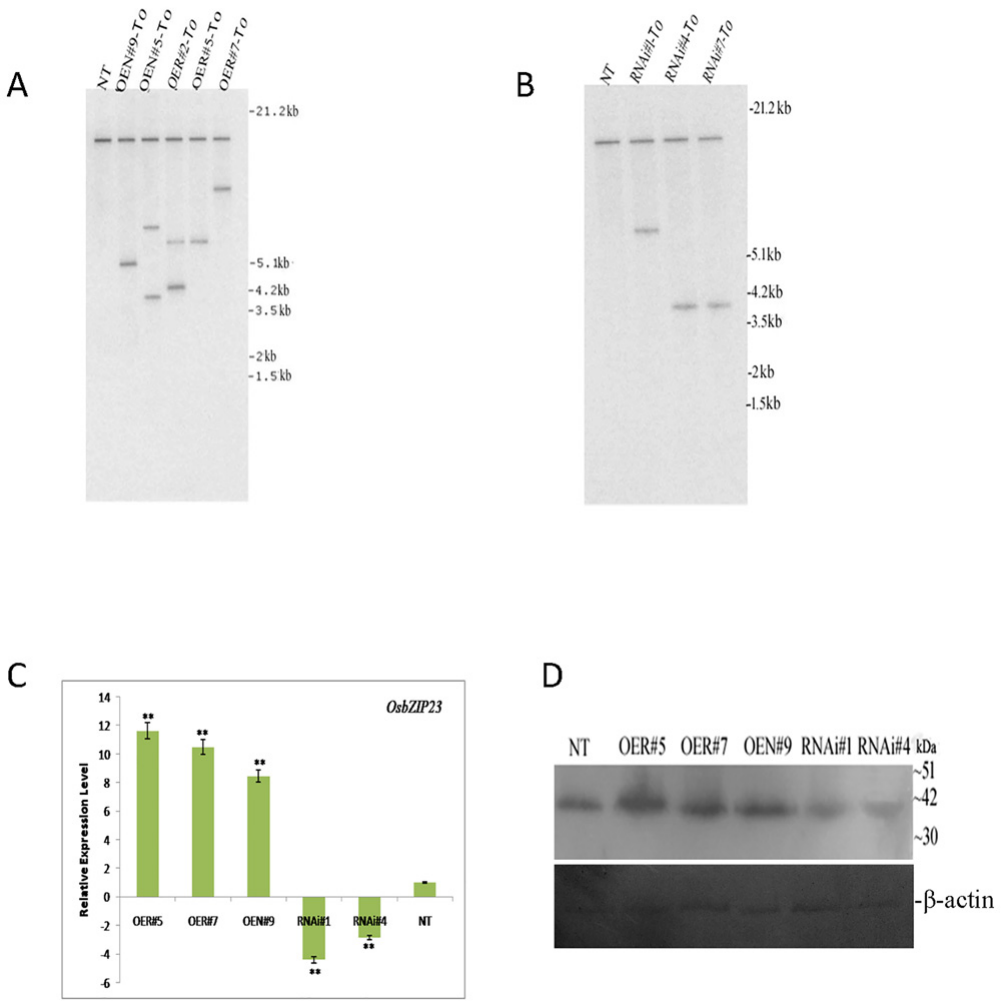
Molecular analyses of transgenic and NT plants for studying the CDS activity of *OsbZIP23* gene. **(A)** Southern blot analysis of T_0_ transformants of OEN and OER developed with the genetic construct of *OsbZIP23* CDS from *O*. *rufipogon* (S3 Fig) and *O*. *nivara* (S4 Fig). The genomic DNA was digested with *Hind*III and probed with the 739 bp *OsbZIP23* CDS. **(B)** Southern blot analysis of T_0_ transformants of RNAi developed with gene silencing construct (S5 Fig) using *Hind*III digested genomic DNA and probed with 739 bp *OsbZIP23* CDS. Lane NT- non-transgenic, Lane M- molecular weight marker. **(C)** Real time PCR analysis showing relative expression level of *OsbZIP23* in three OE lines, two RNAi lines and non-transgenic (NT) plants in vegetative stage, where rice polyubiquitin1 (*OsUbi1*) gene was taken as internal reference. Data bars represent the mean ±SD of triplicate measurement. Statistical analysis of Student’s t test indicated significant differences (* P<0.05, **P<0.01). **(D)** Western blot analysis showing the expression level of OsbZIP23 protein in three OE lines, two RNAi lines and NT plant (upper panel). Equal loading of protein was confirmed by using *β*-actin antibody (lower panel).

To study further the loss-of-function and gain-of-function phenotypes of OsbZIP23 *in planta*, overexpression (OE) and RNAi-mediated silencing (RNAi) of the *OsbZIP23* gene was carried out. For OE, two polymorphic forms of *OsbZIP23* CDS from the wild progenitors- *O*. *rufipogon* and *O*. *nivara* were selected to prepare two different OE constructs, designated as OER and OEN, respectively (S3 and S4 Figs). The polypeptide of *O*. *rufipogon* has a deletion of 5-amino acid residues between the transactivation domain I and domain II of OsbZIP23 (S6 Fig). Similarly for studying gene silencing, an RNAi construct of *OsbZIP23* was also prepared (S5 Fig). The individual genetic construct (OER, OEN and RNAi) was then introduced into the drought-sensitive IR20 rice genotype by *Agrobacterium*-mediated transformation. The tissue culture regenerated OE and RNAi plantlets were screened through hygromycin selection and subjected to Southern blot hybridization using *OsbZIP23* gene-specific probe (Fig 5A and 5B). Three single integration T_1_ OE lines (OER#5, OER#7 and OEN#9) and two single integration T_1_ RNAi lines (RNAi#1 and RNAi#4) along with the control non-transgenic (NT) plants were selected for subsequent studies to examine for the transgene expression and their performance under drought stress. The independent transgenic lines along with NT plants were examined for *OsbZIP23* transcript expression level by real-time PCR analysis (qRT-PCR). The qRT-PCR analysis showed that the relative expression of *OsbZIP23* in OE lines increased significantly (P<0.01), whereas the relative expression level of endogenous *OsbZIP23* in RNAi lines decreased significantly (P<0.01) in comparison to NT plants (Fig 5C). Further, western blot analysis revealed that the abundance of OsbZIP23 protein increased in OE lines and decreased in the RNAi lines compared to NT plants (Fig 5D), supporting the qRT-PCR results.

### Overexpression but not the silencing of *OsbZIP23* gene improves drought tolerance and grain yield in rice

**Fig 6:**
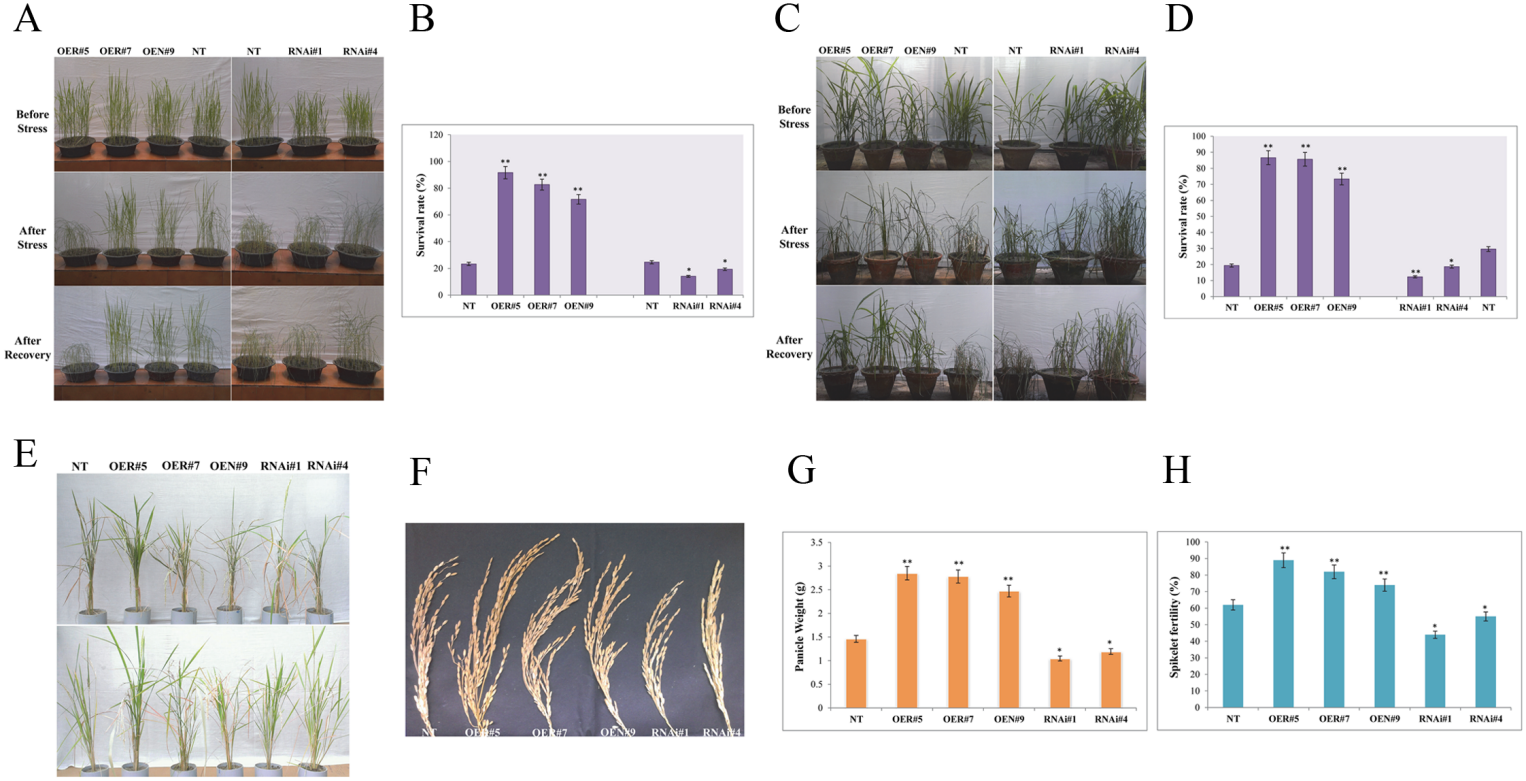
Assessing drought stress tolerance and grain yield of *OsbZIP23* overexpression (OE) and down-regulated (RNAi) transgenic lines. **(A)** Photographs of OE lines, RNAi lines and non-transgenic (NT) plants in the vegetative stage- before and after drought stress and their subsequent recovery after the drought treatment. **(B)** Survival rates of transgenic lines and NT plants, calculated in percentage (%). **(C)** Photographs of OE lines, RNAi lines and NT plants in early reproductive (panicle initiation) stage- before and after drought stress and their subsequent recovery after the drought treatment. **(D)** Survival rates of transgenic lines and NT plants, calculated in %. **(E)** Drought stress in PVC pipes in flowering stage and subsequent recovery till seed maturation stage of OE lines, RNAi lines and NT plants. **(F)** Mature panicle of OE lines, RNAi lines and NT plant. **(G)** Measurement of panicle weight in OE lines, RNAi lines and NT plant. **(H)** Spikelet fertility measurement in OE lines, RNAi lines and NT plants, calculated in %. Data bars represent the mean ±SD of triplicate measurement. Statistical analysis by Student’s t-test indicated significant differences (*P<0.05, ** P<0.01). All the results were based on three independent experiments.

To analyze the tolerance of transgenic lines against drought stress under vegetative and early reproductive (panicle initiation) stages, two set of plant samples (vegetative and reproductive stage) were subjected to drought stress. After the stress period, plants were irrigated for 3 days and survival rate (%) of the OE, RNAi and NT plants was determined. Analysis revealed that the survival rate of OE lines was significantly (P< 0.01) higher than the RNAi lines and NT plants in case of samples at vegetative stage (Fig 6A and 6B) as well as at reproductive stage (Fig 6C and 6D). The decreased drought tolerance of RNAi lines and increased drought tolerance of OE lines in both the stages of plant growth and development suggested that the OsbZIP23 has a crucial role to play in rice plants for adaptation to drought stress. The differences in the period of drought tolerance of OE and RNAi lines prompted us to check the grain yield of these lines under drought stress condition. For this, OE, RNAi and NT plants growing under normal condition in PVC pipes were subjected to progressive drought stress upon attaining the flowering stage. After the stress period, plants were irrigated and allowed to recover at the flowering and seed maturation stage (Fig 6E). It was found that the panicle weight and spikelet fertility of OE lines were significantly (P< 0.01) higher than the RNAi lines and NT plants (Fig 6F, 6G and 6H). The panicle weights of OE lines were ~2.5-3g per panicle, whereas the RNAi lines and NT plants had panicle weighing ~1–1.5g. Similarly, the spikelet fertility was found to be within 75–90% in case of OE lines. On the contrary, the spikelet fertility of RNAi lines was found to be ~40–50%, which was significantly lower than the OE lines. These results indicated that the *OsbZIP23* gene has a significant role to play in drought tolerance and grain yield in rice.

### Transgenic lines overexpressing OsbZIP23 display improved water retention capacity by accumulating more proline and soluble sugars thereby decreasing the cellular oxidative damage cause by drought stress

**Fig 7:**
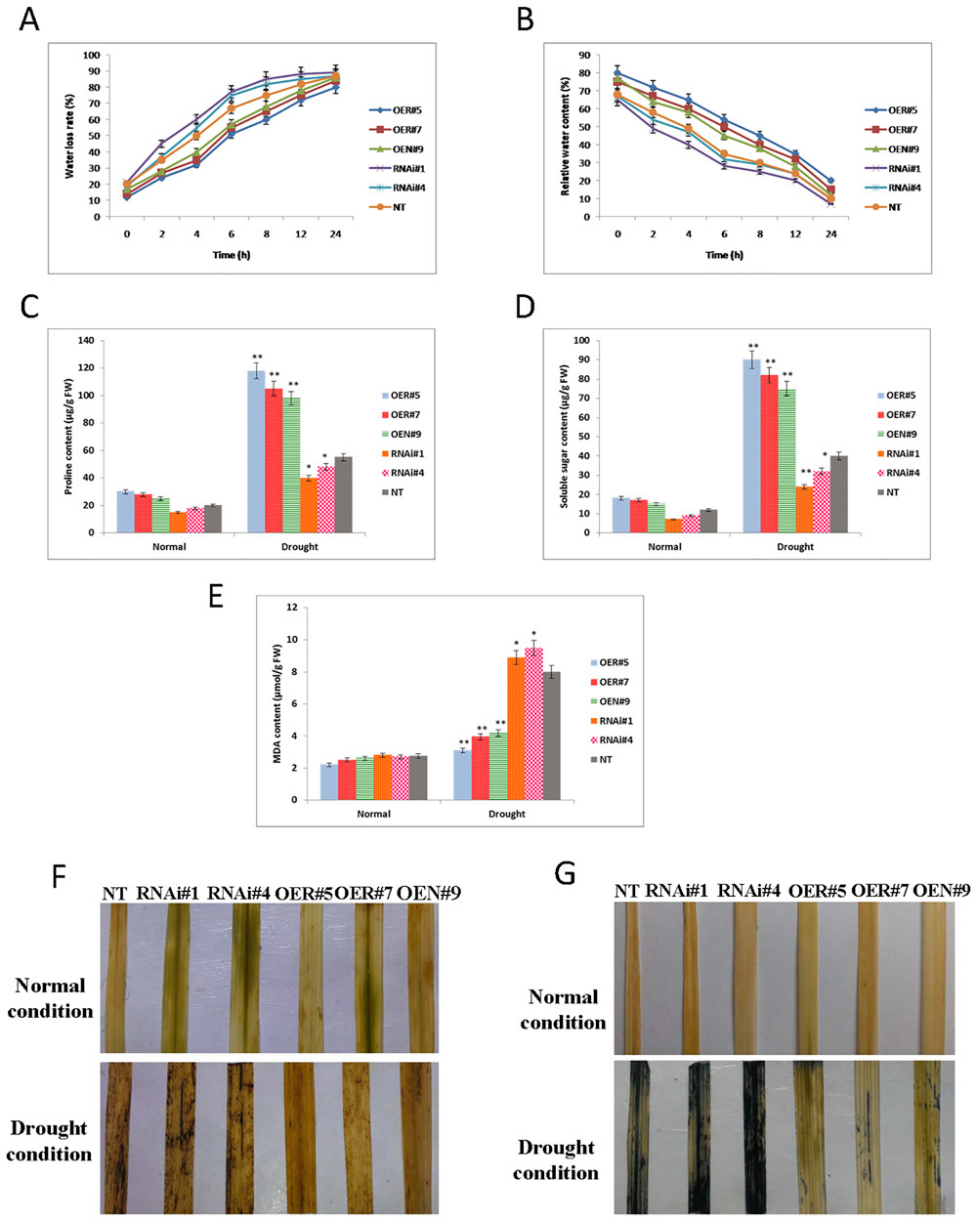
Comparison of leaf water retention capacity and reactive oxygen species (ROS) activity in *OsbZIP23* OE and RNAi lines. **(A)** Water loss rates and **(B)** relative water contents of detached leaves, at the five-leaf stage amongst *OsbZIP23* OE lines, RNAi lines and NT plants. Measurement of **(C)** proline and **(D)** soluble sugar contents in *OsbZIP23* OE lines, RNAi lines and NT plants, after extraction from leaf tissues before and after water stress. All the results were based on three independent experiments. Data bars represent the mean ±SD of triplicate measurement. A statistical analysis by Student’s t-test indicated significant differences (*P<0.05, **P<0.01). **(E)** Measurement of MDA content in *OsbZIP23* OE, RNAi lines and NT plants, after extraction from leaf tissue of rice plants before and after water stress. Data bars represent the mean ±SD of triplicate measurement. A statistical analysis by Student’s t-test indicated significant differences (*P<0.05, **P<0.01). **(F)** Detection of ROS by monitoring H_2_O_2_ production in leaves of *OsbZIP23* OE, RNAi lines and NT plants were visualized by staining with 3, 3^׳^–diaminobenzidine (DAB) under well-watered (normal) and drought stress condition. **(G)** Production of O_2_^−^ ions in leaves of *OsbZIP23* OE, RNAi lines and NT plants were visualized by staining with nitro blue tetrazolium (NBT) under normal and drought stress condition. The results were based on three independent experiments; one set of result is represented here.

The opposite drought tolerance phenotype of OE and RNAi lines further accounted for the analysis of two water retention parameters viz., water loss rate (WLR) and relative water content (RWC). During 0 to 24 hours of dehydration treatment, OE lines exhibited least water loss rate (WLR) and retained the highest relative water content (RWC), while RNAi lines had highest WLR and lowest RWC in comparison to those of NT plants (Fig 7A and 7B). Morphological differences were apparently visible, as the OE leaves largely retained their turgor and the RNAi and NT leaves withered severely. These results suggested that the OsbZIP23 plays a positive role in improving the ability of the plants to retain water under dehydrated conditions. To elucidate the physiological mechanism by which OsbZIP23 improves the water retaining ability of plants under drought stress, proline and soluble sugar contents were determined in the transgenic and NT plants. Under normal growth condition, no significant differences in the contents of proline and soluble sugars were detected amongst OE lines, RNAi lines and NT plants. However, under drought stress condition, the OE lines accumulated dramatically higher contents of proline and soluble sugars, but RNAi lines accumulated smaller amount of these molecules, compared with those in NT plants (Fig 7C and 7D). These findings indicated that the OsbZIP23 helps in water retention by increasing the accumulation of osmolytes, like proline and soluble sugars. Drought stress disrupt the cellular homeostasis of plants by increasing the activity of reactive oxygen species (ROS), which results in high cell toxicity, membrane lipid peroxidation and even cell death. Lipid peroxidation is used as an indicator of oxidative stress. Membrane lipid damage during drought stress was analyzed by measuring the malondialdehyde (MDA) content in OE lines, RNAi lines and NT plants. The results showed that under normal growth condition, there were no significant differences in MDA content amongst OE, RNAi and NT plants. However, under drought stress, lipid peroxidation levels increased in both transgenic lines and NT plants, but the content of MDA in OE lines were significantly (P< 0.01) lower than in the RNAi lines and NT plants (Fig 7E). The decreased level of MDA in the OE lines under drought stress implied that they might be subjected to least serious oxidative damage than the RNAi lines and NT plants. Therefore, it was of interest to detect the accumulation of reactive oxygen species (ROS) in OE, RNAi and NT plants. There were no apparent differences in the level of H_2_O_2_ and O_2_^-^ ions among the leaves of OE, RNAi and NT plants under normal growth conditions after staining with DAB and NBT. However, under drought condition, leaves of RNAi and NT plants exhibited higher level of H_2_O_2_ and O_2_^-^ accumulations than that of OE lines (Fig 7F and 7G), demonstrating that OsbZIP23 helps to minimize the stress-induced oxidative damage *in situ*. These results suggest that transgenic lines overexpressing OsbZIP23 alleviate the effect of drought stress by increasing the accumulation of proline and soluble sugars, which helps the plant to retain more water and avoid the oxidative damage caused by ROS in dehydrating cells.

### The *OsbZIP23* regulates expression of ABA-dependent and stress-responsive genes in transgenic plants

**Fig 8:**
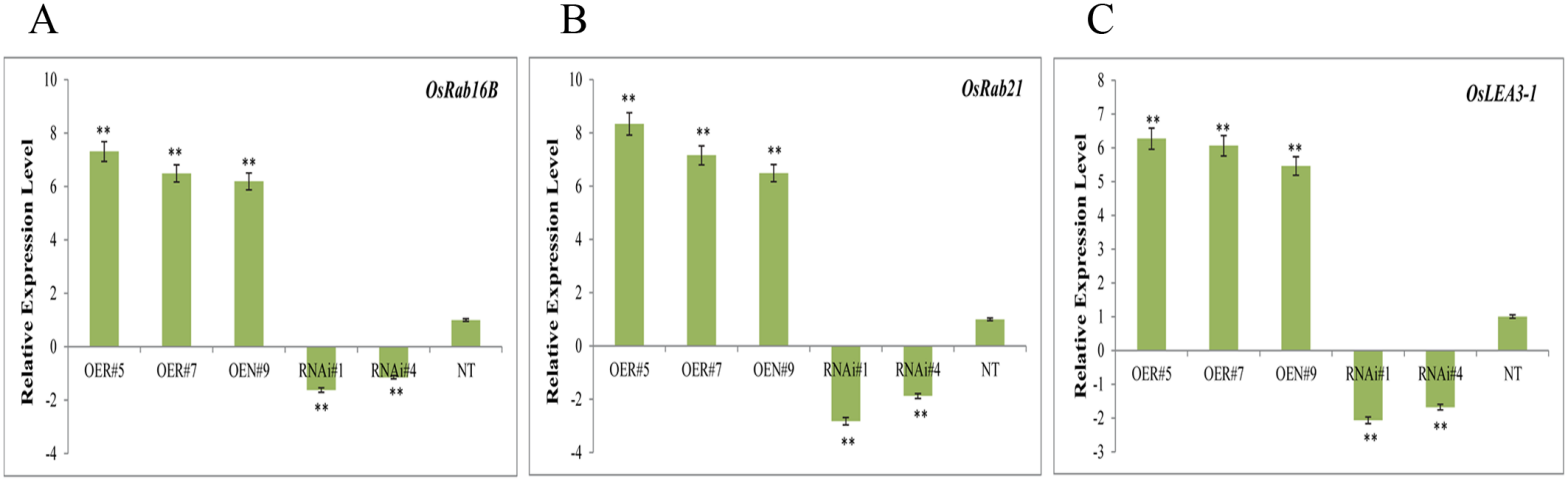
Real time PCR analysis in vegetative stage showing relative expression level of three selected representative drought inducible genes working downstream of *OsbZIP23* in the ABA signaling pathway. **(A)** *OsRab16B*, **(B)** *OsRab21* and **(C)** *OsLEA3*-1 in *OsbZIP23* OE lines, RNAi lines and NT plants, where rice polyubiquitin1 *(OsUbi1)* gene was taken as internal reference. Data bars represent the mean ±SD of triplicate measurement. Statistical analysis by Student’s t-test indicated significant differences (**P<0.01).

To gain deeper understanding of the function of *OsbZIP23* under drought stress, we monitored the transcript levels of three drought-inducible representative genes viz., *OsRab16B*, *OsRab21* and *OsLEA3-1*, working in downstream of *OsbZIP23* in the ABA signaling pathway. The *OsRab16B* and *OsRab21* genes encode dehydrin proteins, which act by decreasing cellular osmotic potential; whereas the *OsLEA3-1* encodes late embryogenesis abundant (LEA) protein. The relative expression level of Os*Rab16B*, *OsRab21* and *OsLEA3-1* in vegetative stage were significantly (P< 0.01) increased in OE lines than in the NT plants, as revealed through qRT-PCR analysis (Fig 8A, 8B and 8C). On the contrary, the relative expression levels of these three genes were significantly (P< 0.01) lower in RNAi lines compared to NT plants (Fig 8A, 8B and 8C). Further, we analysed the relative expression level of these three genes along with *OsbZIP23* in the reproductive stage. The result showed similar trend of increased expression in OE lines and decreased expression in RNAi lines in comparison to NT plants (S7 Fig). These findings suggested that the *OsbZIP23* overexpression activates but down-regulation affects the transcription of hierarchically downstream genes involved in ABA signaling and stress responses.

### ABA sensitivity differs in *OsbZIP23* OE and RNAi lines at germination and post-germination stages

**Fig 9:**
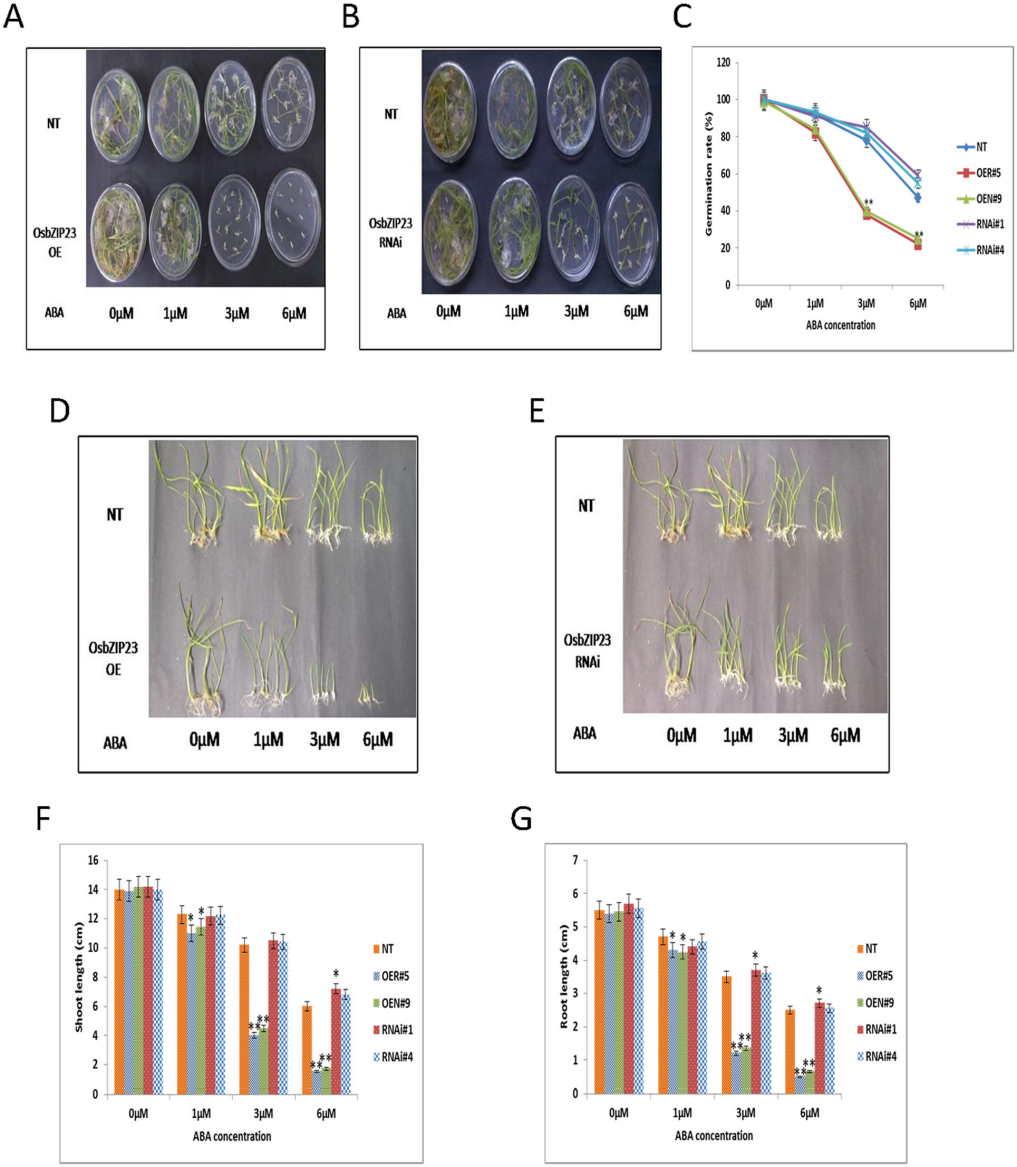
Evaluating ABA sensitivity of *OsbZIP23* OE and RNAi lines at germination and post-germination stages. Germination performance of seeds from **(A)** *OsbZIP23* OE lines (OER#5, OEN#9), and **(B)** *OsbZIP23* RNAi lines (RNAi#1, RNAi#4) in comparison to NT plants on MS agar medium containing 0, 1, 3 and 6 μM ABA at 10^th^ day. **(C)** Calculation of the germination rates (%) of *OsbZIP23* OE, RNAi and NT seeds. **(D** and **E)** Performance of OE, RNAi and NT seedlings in ½ MS liquid medium containing 0, 1, 3 and 6 μM of ABA. Measurement of (**F**) shoot length and **(G)** root length of OE, RNAi and NT seedlings grown on different concentrations of ABA after 14 days. Data bars represent the mean ±SD of triplicate measurement. Statistical analysis by Student’s t-test indicated significant differences (*P<0.05, ** P<0.01). All the results were based on three independent experiments.

To check the ABA sensitivity of rice transgenic lines, seeds of two OE lines (OER#5 and OEN#9), two RNAi lines (RNAi#1 and RNAi#4) and NT plants were germinated on MS agar medium in different concentration of ABA (0, 1, 3 and 6 μM) for 10 days. The germination rate of OE lines had no significant difference with RNAi lines and NT plants at 0 and 1μM ABA, but it was significantly (P< 0.01) lower in OE lines than that of the RNAi lines and NT plants at 3 and 6 μM ABA (Fig 9A, 9B and 9C), suggesting that the ABA sensitivity was increased in *OsbZIP23* OE lines at germination stage. The ABA sensitivity of transgenic plants was investigated at the post-germination stage. It was observed that at 0 μM concentration of ABA, the shoots and roots of OE seedlings (grown for 2 weeks) showed no significant morphological differences with RNAi lines and NT plants. However, the length of shoots and roots of OE lines exhibited significant disparity in comparison to RNAi lines and NT plants at 1, 3 and 6 μM ABA (Fig 9D, 9E, 9F and 9G). These results indicated that the overexpression but not the down-regulation of *OsbZIP23* had increased ABA sensitivity at the post-germination stage, similar to the situation at germination stage.

## Discussion

Progress in plant breeding has made possible the accumulation of superior alleles from vast plant genetic resources for generation of improved and high yielding varieties of agricultural crops. However, these superior alleles were not utilized properly as these were left behind during selective evolution and domestication carried out by farmers and breeders. These untapped genetic variations existing in wild relatives and land races of crop plants could be explored for development of superior cultivars [44]. Allele mining is a well-established approach to dissect naturally existing allelic variations of candidate genes controlling key agronomic traits, which have potential applications in crop improvement. Nucleotide polymorphism in the promoter region or in the coding DNA sequence can have dramatic effects on the expression of the candidate gene or the functional activity of the corresponding protein. The consequences of these effects account for the attainment of desirable or undesirable agronomic traits. Although a number of genes have been reported to be involved in the adaptation of various abiotic stresses in plants, the allelic diversity of these genes is poorly understood [45]. To cope with drought stress, it is of vital importance to understand the role of naturally occurring allelic variants of known genes working in stress responsive pathways leading to plant’s tolerance for successful growth and development [46].

We investigated the allelic polymorphism of *OsbZIP23* gene that encodes a crucial transcription factor in drought stress adaptation pathway in nine selected *indica* rice genotypes of drought-tolerant and drought-sensitive cultivars along with two wild progenitors of rice and four other representative members of the Poaceae family. Our study revealed the presence of non-synonymous single nucleotide polymorphisms (SNPs) at four places in the coding DNA sequence (CDS) and a deletion of 15 nucleotides at one place in the CDS of this gene (Fig 1B). The first two non-synonymous SNPs result in a non-polar amino acid substitution (F to L) at the 202^nd^ position and a positively charged amino acid substitution (K to N) at the 263^rd^ position in some of the genotypes (Fig 1B). The 3^rd^ non-synonymous SNP was observed in *OsbZIP23* CDS of bajra, where a polar amino acid (Q) at the 15^th^ position changes to the non-polar amino acid (L). The 4^th^ non-synonymous SNP was observed in the *OsbZIP23* CDS of *O*. *nivara* and sorghum, where a non-polar amino acid substitution (A to V) occurs at the 86^th^ position of the protein. In wild rice progenitor *O*. *rufipogon*, which is a drought-tolerant genotype, a major 15-nucleotide deletion in the CDS of *OsbZIP23* was observed; eliminating the amino acid sequence AAEHA (or AVEHA compared to *O*. *nivara* and sorghum) from the position 85^th^ to 89^th^ at the N-terminal region (Fig 1B). This sequence of five amino acids is normally located specifically between the transactivation domain I and domain II of the OsbZIP23 polypeptide (S6 Fig). Out of the deleted five amino acids (AAEHA), three were non-polar (A) residues, one negatively charged (E) residue and one positively charged (H) residue. The 5-amino acid deletion of *O*. *rufipogon* is not observed in any other rice genotypes, and this may be correlated to the fact that all are *indica* sub-types that are derived from *O*. *nivara* progenitor. Similar reports of non-synonymous SNPs and deletions leading to alteration of functions of some other genes have been reported previously [45, 47].

Presence of allelic polymorphism in the CDS of *OsbZIP23* gene in the selected drought-tolerant and drought-sensitive rice genotypes as well as wild rice progenitors prompted us to further demonstrate the relative expression level of this gene in all the rice genotypes through qRT-PCR analyses. To know which plant part to select for analysis of these 11 rice genotypes, at first we investigated different tissues from one drought-tolerant genotype, Vandana. The expression of *OsbZIP23* was found to be tissue specific, with the highest expression level in leaf followed by leaf sheath (Fig 2A). Hence leaf tissues were used for all subsequent analyses of *OsbZIP23* gene expression. The relative expression level of *OsbZIP23* in the selected rice genotypes was found to be significantly variable during before stress (BS), after stress (AS) and after recovery (AR) conditions in both vegetative and reproductive stages (Fig 2B and 2C). The relative expression levels of *OsbZIP23* under vegetative and reproductive stages were found to be much higher in drought-tolerant genotypes, compared to the drought-sensitive ones. It was also observed that the relative expression level of *OsbZIP23* in all the rice genotypes was consistently higher in reproductive stage as compared to vegetative stage (Fig 2B and 2C), which signifies the important role of *OsbZIP23* in drought adaptation of plants at the reproductive stage. The variation in the relative expression level of *OsbZIP23* in the selected rice genotypes further guided us to verify the copy number of endogenous *OsbZIP23* in the rice genotypes through Southern blot analysis, which revealed the single copy of endogenous *OsbZIP23* gene in all the rice genotypes tested (Fig 2D). Presence of single copy of the endogenous *OsbZIP23* gene in all the rice genotypes suggests that the copy number has no effect on variable relative expression level of *OsbZIP23* in drought-tolerant and drought-sensitive rice genotypes.

The findings that all the drought-tolerant rice genotypes express the *OsbZIP23* gene at a much higher level than drought-sensitive genotypes and there is no anticipated copy number effect, directed us to dig into the promoter regions of this gene in different rice genotypes. We selected one drought-tolerant (wild rice *O*. *rufipogon*) and one drought-sensitive (*O*. *sativa* cultivar IR20) rice genotypes to isolate, clone and characterize the promoter region through *GFP* reporter gene expression. The prediction based analysis of both the isolated promoter sequences revealed the presence of various stress responsive *cis*-acting elements (Fig 3B). The number of these *cis*-regulatory elements varies within these two promoter sequences. One striking observation is that the *OsbZIP23* promoter of IR20 genotype carries a deletion of a sequence of 35 nucleotides in comparison to *O*. *rufipogon* promoter at the 5'-UTR region. Within this 35-nucleotide deletion, two extra MYB-binding elements are found to be present in *O*. *rufipogon*. Interestingly, the *OsbZIP23* promoter is TATA-less, however Y- patches are found to be present around transcription start site (TSS). Y-patches are the core promoter elements, which are direction-sensitive pyrimidine rich boxes found around TSS [48–49]. The variation in the number of *cis*-acting elements as well as a deletion of 35-nucleotide sequence prompted us to check the functional activity of both the promoters by expression analysis of the GFP reporter gene using the promoter-probe constructs (S1 and S2 Figs). Analysis of transgenic leaf tissues revealed that both the fluorescence intensity (Fig 4B) and the relative expression level (Fig 4C) of GFP are much higher in RuP#2 and RuP#3 lines than the 20P#1 and 20P#2 lines under drought stress condition. These results suggest that the promoter activity of *O*. *rufipogon* is comparatively higher than the IR20 rice genotype, which could be a reason that under similar growth condition, the expression of *OsbZIP23* gene varies significantly in drought-tolerant and drought-sensitive genotypes.

We have cloned and sequenced the *OsbZIP23* CDS from the six drought-sensitive and five drought-tolerant rice genotypes. The detailed sequence analysis of the derived polypeptides revealed no striking differences, except the deleted 5-amino acid sequence (AAEHA) in *O*. *rufipogon*, which is normally present in all other rice genotypes (except *O*. *nivara* which has AVEHA in place of AAEHA) between the transactivation domain I and domain II of OsbZIP23 (S6 Fig). Therefore, we wanted to examine the variation on functional activity, if any, between the two natural allelic variants of *OsbZIP23* CDS from two wild rice progenitors, i.e., *O*. *nivara* (having 1083 bp CDS) and *O*. *rufipogon* (having 1068 bp CDS with deleted 15 nucleotides encoding AVEHA in comparison to *O*. *nivara*). The respective CDS was transgenically expressed in a drought-sensitive cultivar (IR20) using the constitiutive *OsUbi1* promoter driven expression cassette (S3 and S4 Figs). Further to compare the overexpression of these two CDS in transgenic rice lines (OEN and OER), RNAi-mediated *OsbZIP23* gene silencing transgenic lines (RNAi) were also developed with an appropriate genetic construct (S5 Fig). The overexpression (OE) and RNAi-mediated silencing (RNAi) of *OsbZIP23* gene in drought-sensitive *indica* rice genotype IR20 resulted in expected increase and decrease of *OsbZIP23* expression in OE lines (OEN#9, OER#5 and OER#7) and RNAi lines (RNAi#1and RNAi#4), respectively in comparison to non-transgenic (NT) plants at both transcriptional (Fig 5C) and translational levels (Fig 5D). Under the drought stress condition, the OE and RNAi transgenic lines showed opposite phenotypes. In both vegetative and reproductive stages, the OE lines exhibited increased drought tolerance in terms of higher survival rate than the RNAi lines and NT plants; whereas the RNAi lines performed significantly poorer than the NT plants under drought stress condition (Fig 6A, 6B, 6C and 6D). Analysis of yield attributing traits revealed that the OE lines had increased panicle weight and spikelet fertility in comparison to RNAi lines and NT plants under drought stress (Fig 6E, 6F, 6G and 6H). These results clearly established that the transgenic overexpression of *OsbZIP23* improves drought tolerance and grain yield in rice. On the contrary, down-regulation of the endogenous *OsbZIP23* makes the rice plants drought sensitive and poor yielder. Previous works on overexpression or silencing of ABA-responsive genes have documented similar results [39, 50]. The ability to retain water is a crucial factor for plants to combat drought. The water loss rate (WLR) and relative water content (RWC) are the two important parameters that reflect the water status of the plant [39, 51]. Measurement of WLR and RWC in leaf tissues revealed that the OE lines had highest water retention capacity compared to RNAi lines and NT plants (Fig 7A and 7B). This finding strongly indicates that the *OsbZIP23* is involved in maintaining the ability of plants to retain water. We further investigated the physiological mechanism by which *OsbZIP23* enables the plant to retain water and mitigate the effect of drought stress. It is established that under water-limiting condition, proline and sugars are accumulated in plant cells, resulting decrease in the cellular osmotic potential [52]. Our results documented that the content of both proline and soluble sugars in the leaf tissues of *OsbZIP23* OE lines was much higher than the RNAi lines and NT plants under drought stress condition (Fig 7C and 7D). Drought stress can reduce photosynthesis, and results in accumulation of excess reactive oxygen species (ROS), which leads to cell toxicity, membrane peroxidation and even cell death [53]. There is a constant need for efficient mechanisms to avoid oxidative damage to cell. Malondialdehyde (MDA) is a stress marker that measures the degree of damage caused by stress in plants [54]. Our analysis on transgenic lines displaying lower MDA content in OE lines in comparison to RNAi lines and NT plants under drought stress (Fig 7E) implies that the drought stress-induced oxidative damage causes less harm to OE lines than RNAi lines and NT plants. Therefore, accumulation of ROS under drought stress in OE lines, RNAi lines and NT plants were checked. Accumulation of peroxide and superoxide free radicals in leaf tissues of OE lines was negligible in comparison to RNAi lines and NT plants under drought stress condition (Fig 7F and 7G). It can be inferred that the transgenically overexpressed *OsbZIP23* has improved drought tolerance by ROS detoxification, and thereby leading to less membrane damage, which indeed resulted from increased water retention through accumulation of compatible osmolytes.

To understand the molecular mechanism behind the OsbZIP23-mediated drought stress adaptation, we analyzed the transcript level of three representative inducible marker genes of ABA signaling pathway viz., *OsRab16B*, *OsRab21* and *OsLEA3-1*. Rabs are dehydrin family proteins reported to decrease the cellular osmotic potential while protecting the plants under drought stress [55]. LEA or late embryogenesis abundant proteins have been proposed to maintain the cell membrane structure stable under water deficit condition by reducing the damage caused by high concentration of ions in dehydrating cells [56], and are regulated by bZIP transcription factors through ABRE *cis*-elements [26]. The increased and decreased expression of *OsRab16B*, *OsRab21* and *OsLEA3-1* genes in OE and RNAi lines, respectively (Fig 8 and S7 Fig) suggests that the *OsbZIP23* is a transcriptional activator of these genes in rice. The high expression level of these genes, in turn, most likely leads to up-regulation of other genes that are controlled by OsRab16B and OsRab21 and OsLEA3-1 in an ABA-dependent manner, and could be explained by the established ABA signaling pathway (S8 Fig) [57]. It is well known that the phytohormone ABA maintains seed dormancy, inhibits seed germination and retards seedling growth [39]. Drought stress induces ABA biosynthesis and triggers ABA-dependent signaling pathway. Thus, we investigated ABA-sensitivity of OE and RNAi lines, which displayed significantly increased and decreased sensitivity, respectively to exogenous ABA at both germination and post-germination stages (Fig 9). These results are consistent with the previous observations on increased ABA sensitivity of the transgenic rice lines overexpressing *OsbZIP23* gene [25], confirming that the ABA-signaling pathway is positively regulated by the OsbZIP23 transcription factor.

## Conclusions

Allelic polymorphism analysis in *OsbZIP23* coding DNA sequence showed presence of some non-synonymous SNPs in different genotypes as well as a major 5-amino acid deletion in *O*. *rufipogon*. The *OsbZIP23* transcript expression profiling under vegetative and reproductive stages revealed comparatively higher expression level of *OsbZIP23* in drought-tolerant genotypes than the drought-sensitive ones. The *OsbZIP23* promoter of drought-tolerant *O*. *rufipogon* showed higher promoter activity than the drought-sensitive IR20 rice genotype. Overexpression of either of the two polymorphic forms of *OsbZIP23* from wild rice genotypes and endogenous *OsbZIP23* gene silencing through RNAi revealed that the enhanced expression of *OsbZIP23* improves drought tolerance and grain yield in drought-sensitive rice genotype. Further the overexpression lines exhibited increase in water retention capacity through higher accumulation of compatible osmolytes, leading to the decrease in membrane damage caused by the activity of reactive oxygen species. The drought tolerance and yield improvement in rice genotypes are found to be dependent on the enhanced gene expression of *OsbZIP23* due to higher promoter activity rather than the natural polymorphism in coding sequence. Therefore, the present study advances our understanding on developing drought-tolerant elite rice cultivars utilizing highly expressed promoter to drive the *OsbZIP23* transgene. The information generated through the present work will help us to develop gene-based molecular marker for selective breeding to improve drought tolerance in rice crop utilizing this type of transcriptional regulator.

## Supporting Information

S1 Fig Schematic representation of *OsbZIP23* promoter-*GFP* reporter gene construct in pCAMBIA1300 plasmid. Promoter sequence of 1612 bp from drought tolerant wild genotype *O*. *rufipogon* was fused to *GFP* reporter gene and the transgenic lines developed with this genetic construct were designated as RuP series.
(TIF)

S2 Fig Schematic representation of *OsbZIP23* promoter-*GFP* reporter gene construct in pCAMBIA1300 plasmid. Promoter sequence of 1578 bp from drought sensitive *indica* rice genotype IR20 was fused with *GFP* reporter gene and the transgenic lines developed with this expression cassette were designated as 20P series.
(TIF)

S3 Fig Schematic representation of the overexpression (OE) construct of 1068 bp CDS of *OsbZIP23* from *O*. *rufipogon* in pCAMBIA1300 plasmid. The transgenic rice lines developed with this genetic construct were designated as OER series.
(TIF)

S4 Fig Schematic representation of the overexpression (OE) construct of 1083 bp CDS of *OsbZIP23* from *O*. *nivara* in pCAMBIA1300 plasmid. The transgenic rice lines developed with this genetic construct were designated as OER series.
(TIF)

S5 Fig Schematic representation of the RNAi-mediated silencing (RNAi) construct of *OsbZIP23* gene in pCAMBIA1300 plasmid. The 399 bp of *OsbZIP23* CDS (3'-part) from *O*. *rufipogon* was cloned in sense and antisense direction containing an arbitrary 200 bp linker. The transgenic rice lines developed with this genetic construct were designated as RNAi series.
(TIF)

S6 Fig Structural domain prediction of OsbZIP23 protein using ScanProsite web tool (http://prosite.expasy.org/scanprosite). In both drought tolerant genotypes **(A)** *O*. *rufipogon* and **(B)** *O*. *nivara*, three conserved transactivation domains (domain I, II and III) and one basic leucine zipper (bZIP) domain was predicted. Presence of three protein kinase C phosphorylation site (red pointed serine and threonine), five casein kinase II phosphorylation site (underlined threonine residues) and 11 N-myristoylation sites (indicated in blue colour) were also predicted. The 5-amino acid (AVEHA) deletion in *O*. *rufipogon* is indicated with gap. Note that this 5-amino acid sequence is AAEHA in all other genotypes tested except *O*. *nivara* and sorghum, which have AVEHA (Fig 1B).
(TIF)

S7 Fig Real time PCR analysis in reproductive stage showing relative expression level of *OsbZIP23* and three selected representative drought inducible genes working downstream in the ABA signaling pathway. **(A)** *OsbZIP23* **(B)** *OsRab16B* **(C)** *OsRab21*, and **(D)** *OsLEA3*-1 in *OsbZIP23* OE lines, RNAi lines and NT plants, where rice polyubiquitin1 *(OsUbi1)* gene was taken as internal reference. Data bars represent the mean ±SD of triplicate measurement. Statistical analysis by Student’s t-test indicated significant differences (**P<0.01).
(TIFF)

S8 Fig Schematic representation of the ABA signaling pathway under drought stress. Drought induces accumulation of ABA, which is regulated by PARP and LOS5. ABA is perceived by the PYR1 receptor and induces the expression of SnRK2 kinases by releasing ABI1 phosphatase. This in turn activates the downstream AREB/bZIP-transcription factors. The activated AREBs (through the interaction with ABRE *cis*-elements) induce the expression of a series of downstream genes to produce different classes of osmoprotectants, which protects the plant by decreasing cellular osmotic potential. Adopted from Yang et al., 2010.
(TIFF)

S1 Table List of primers used in this study. (DOCX)
