# Correction: Enhanced Gene Expression Rather than Natural Polymorphism in Coding Sequence of the *OsbZIP23* Determines Drought Tolerance and Yield Improvement in Rice Genotypes

**DOI:** 10.1371/journal.pone.0187172

**Published:** 2017-10-23

**Authors:** Avishek Dey, Milan Kumar Samanta, Srimonta Gayen, Soumitra K. Sen, Mrinal K. Maiti

During the preparation of this article for publication, an error was introduced into Fig 6A. The left-most column in this figure should be labeled NT and the following three columns should be labeled OER#5, OER#7 and OEN#9 in that order. The corrected figure is included here.

**Fig 6:**
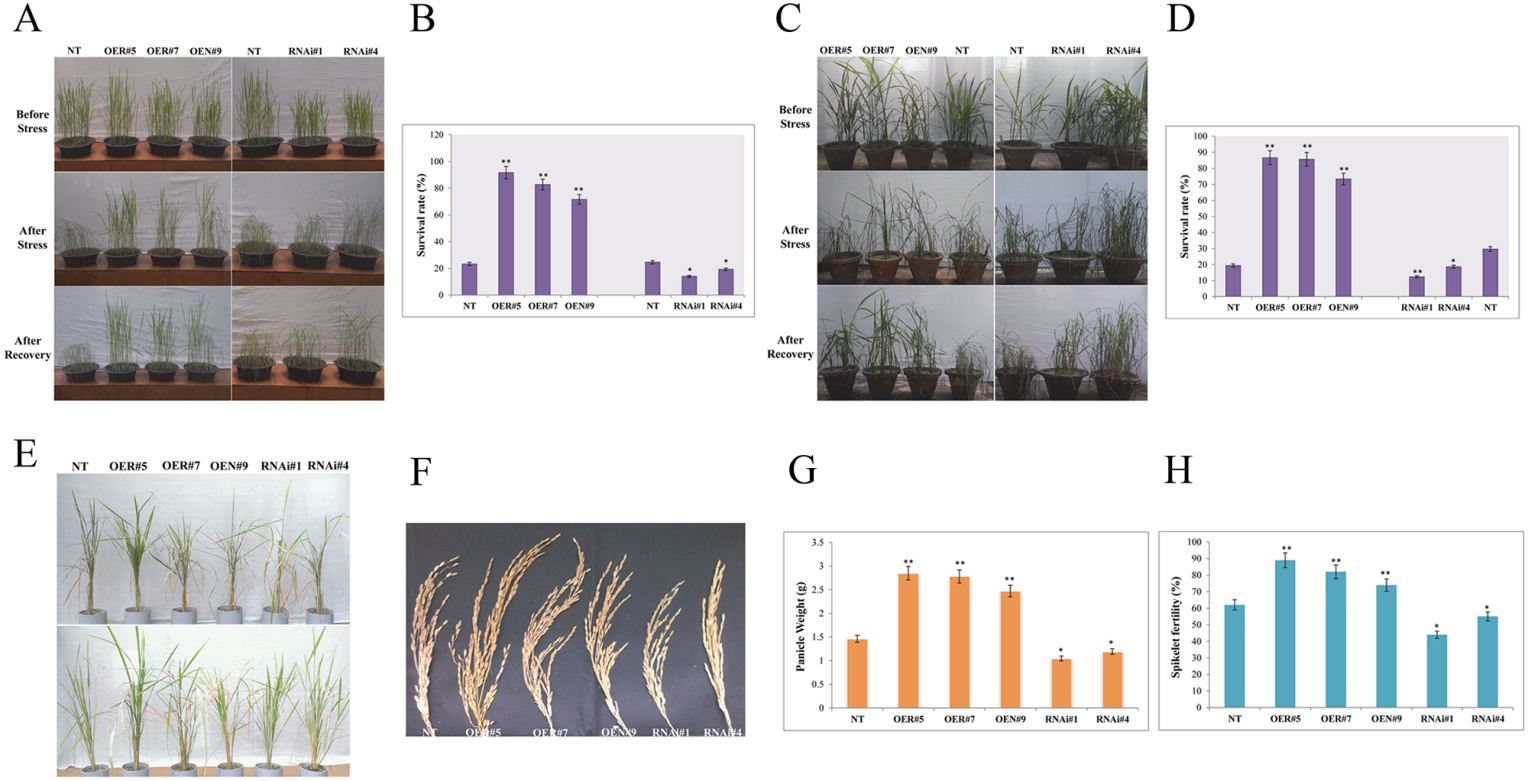
Assessing drought stress tolerance and grain yield of *OsbZIP23* overexpression (OE) and down-regulated (RNAi) transgenic lines. **(A)** Photographs of OE lines, RNAi lines and non-transgenic (NT) plants in the vegetative stage- before and after drought stress and their subsequent recovery after the drought treatment. **(B)** Survival rates of transgenic lines and NT plants, calculated in percentage (%). **(C)** Photographs of OE lines, RNAi lines and NT plants in early reproductive (panicle initiation) stage- before and after drought stress and their subsequent recovery after the drought treatment. **(D)** Survival rates of transgenic lines and NT plants, calculated in %. **(E)** Drought stress in PVC pipes in flowering stage and subsequent recovery till seed maturation stage of OE lines, RNAi lines and NT plants. **(F)** Mature panicle of OE lines, RNAi lines and NT plant. **(G)** Measurement of panicle weight in OE lines, RNAi lines and NT plant. **(H)** Spikelet fertility measurement in OE lines, RNAi lines and NT plants, calculated in %. Data bars represent the mean ±SD of triplicate measurement. Statistical analysis by Student’s t-test indicated significant differences (*P<0.05, ** P<0.01). All the results were based on three independent experiments.

In response to concerns raised by readers, the authors would like to further clarify that they duplicated NT panels in Fig 9A, 9B, 9D and 9E. The panels were duplicated to represent the observed phenotype of NT plants, which was the same in all three replicate plates examined in this study and was used to compare separately with the OE and RNAi lines in the respective panels. Three of the dishes in Fig 9B reproduce dishes in Fig 9A, although they were rotated in error during the preparation of the figure. The authors apologize that this duplication was not clearly described in the figure legend. The authors have provided a corrected Fig 9 with identical NT rows in Fig 9A and 9B.

**Fig 9:**
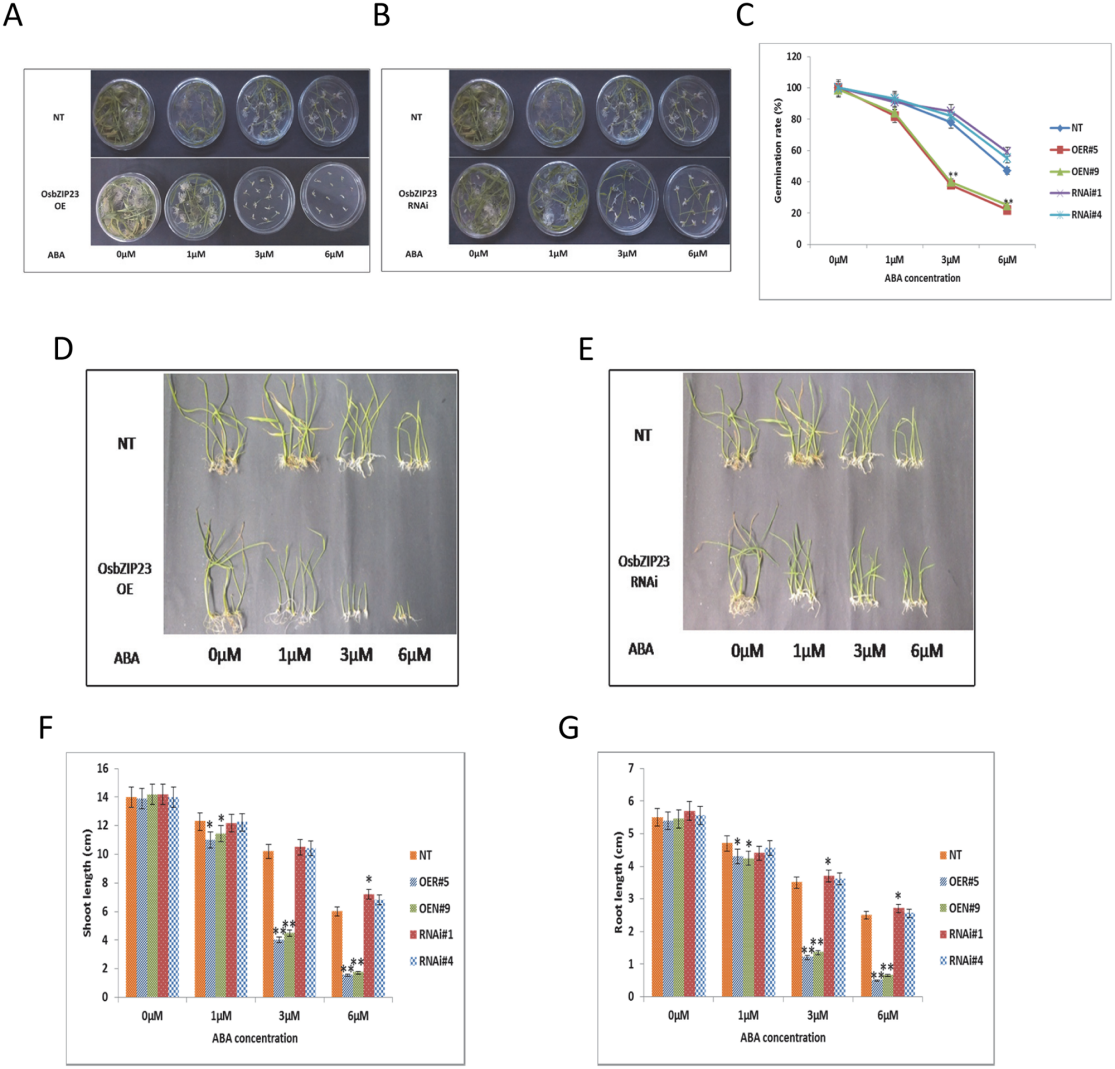
Evaluating ABA sensitivity of *OsbZIP23* OE and RNAi lines at germination and post-germination stages. Germination performance of seeds from **(A)** *OsbZIP23* OE lines (OER#5, OEN#9), and **(B)** *OsbZIP23* RNAi lines (RNAi#1, RNAi#4) in comparison to NT plants on MS agar medium containing 0, 1, 3 and 6 μM ABA at 10^th^ day. **(C)** Calculation of the germination rates (%) of OsbZIP23 OE, RNAi and NT seeds. **(D and E)** Performance of OE, RNAi and NT seedlings in ½ MS liquid medium containing 0, 1, 3 and 6 μM of ABA. Measurement of **(F)** shoot length and **(G)** root length of OE, RNAi and NT seedlings grown on different concentrations of ABA after 14 days. For representation and better comparison, the NT panels are duplicated in A-B and D-E to represent the observed phenotype of NT plants, which was the same in all three replicate plates, examined in this study and was used to compare separately with the OE and RNAi lines in the respective panels. Data bars represent the mean ±SD of triplicate measurement. Statistical analysis by Student’s t-test indicated significant differences (*P<0.05, ** P<0.01). All the results were based on three independent experiments.

The Data Availability statement for this paper is incorrect. The correct statement is: All relevant data are available on figshare (URL: https://doi.org/10.6084/m9.figshare.4509008).
