# Supplementary figures and images for: Enhanced Gene Expression Rather than Natural Polymorphism in Coding Sequence of the OsbZIP23 Determines Drought Tolerance and Yield Improvement in Rice Genotypes
Source: PLoS One. 2016 Mar 9;11(3):e0150763. doi: 10.1371/journal.pone.0150763 (PMC4784890; doi:10.1371/journal.pone.0150763)

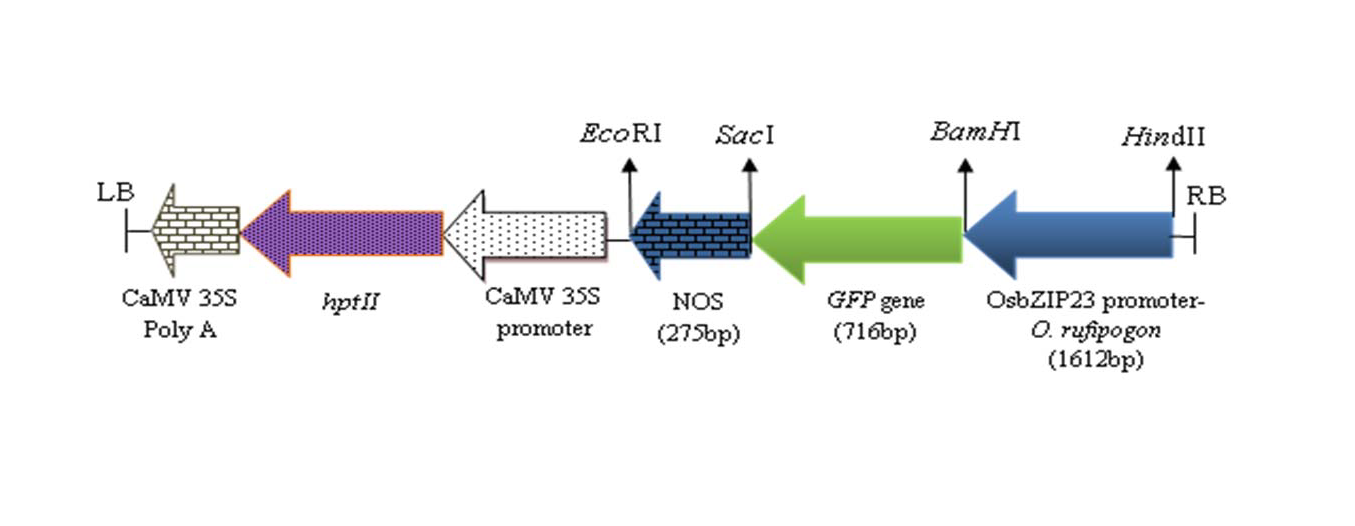

Supplement: S1 Fig — Promoter sequence of 1612 bp from drought tolerant wild genotype O. rufipogon was fused to GFP reporter gene and the transgenic lines developed with this genetic construct were designated as RuP series. (TIF) [file pone.0150763.s001.tif]

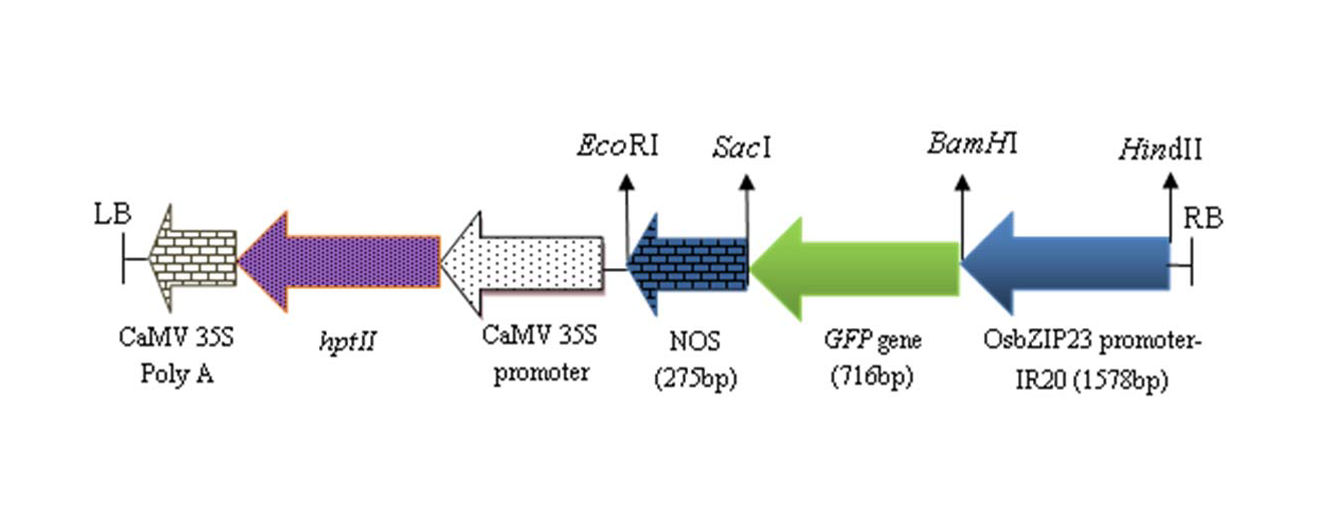

Supplement: S2 Fig — Promoter sequence of 1578 bp from drought sensitive indica rice genotype IR20 was fused with GFP reporter gene and the transgenic lines developed with this expression cassette were designated as 20P series. (TIF) [file pone.0150763.s002.tif]

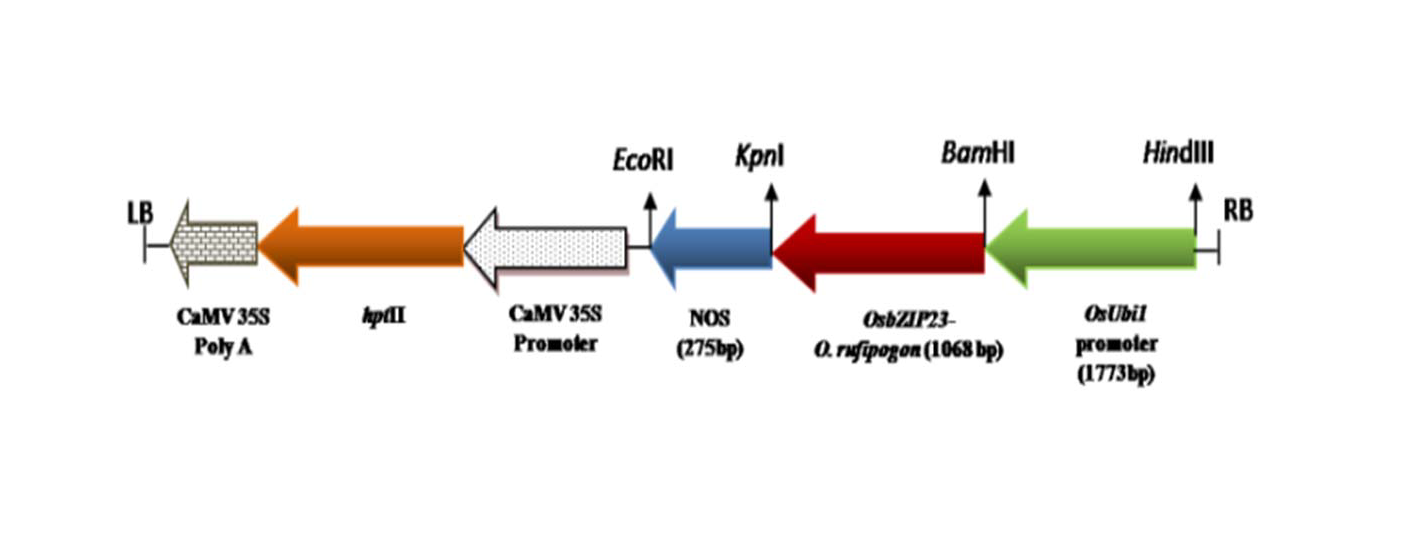

Supplement: S3 Fig — The transgenic rice lines developed with this genetic construct were designated as OER series. (TIF) [file pone.0150763.s003.tif]

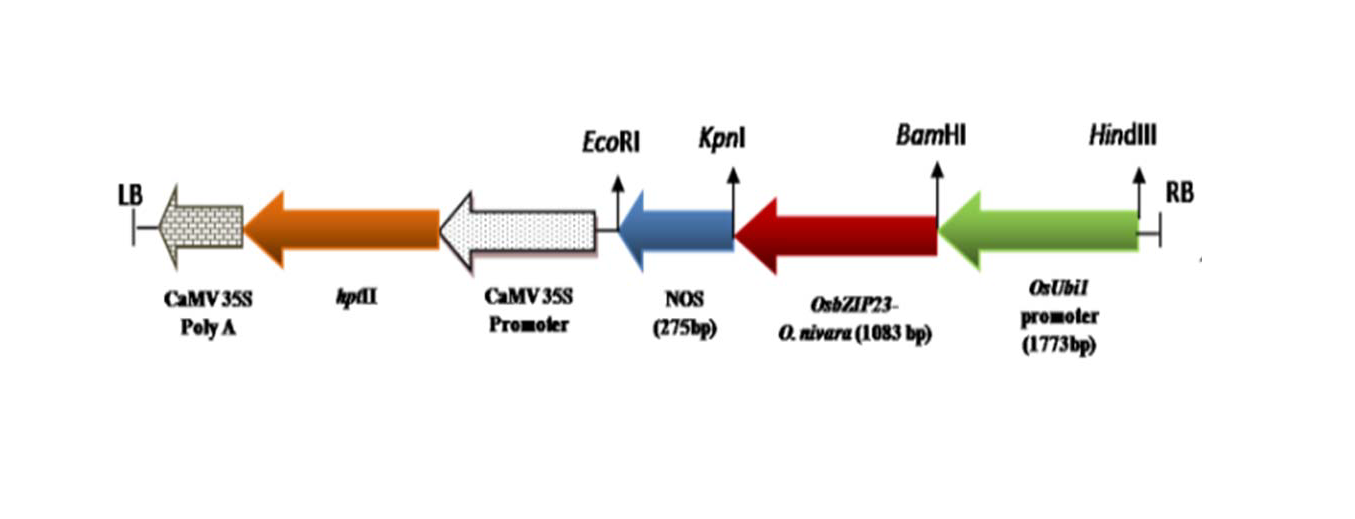

Supplement: S4 Fig — The transgenic rice lines developed with this genetic construct were designated as OER series. (TIF) [file pone.0150763.s004.tif]

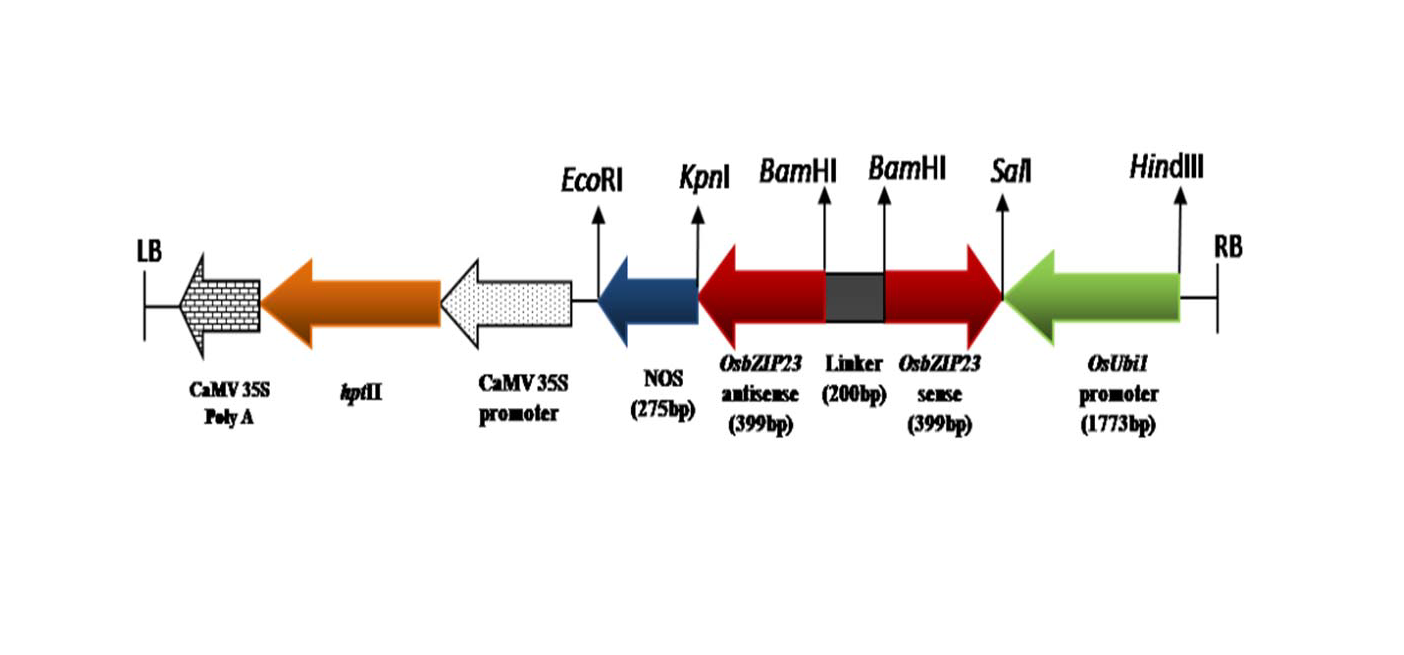

Supplement: S5 Fig — The 399 bp of OsbZIP23 CDS (3'-part) from O. rufipogon was cloned in sense and antisense direction containing an arbitrary 200 bp linker. The transgenic rice lines developed with this genetic construct were designated as RNAi series. (TIF) [file pone.0150763.s005.tif]

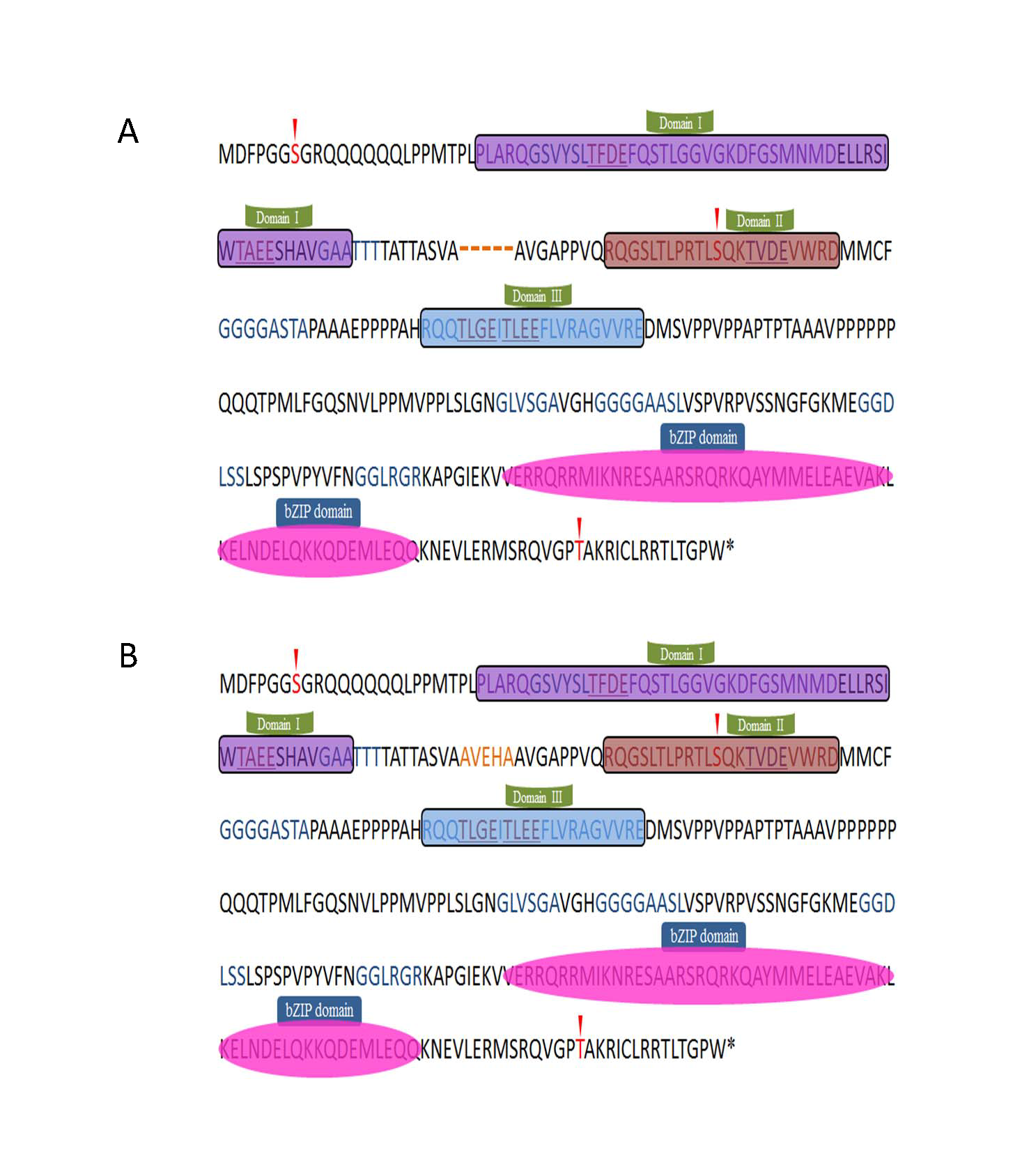

Supplement: S6 Fig — In both drought tolerant genotypes (A) O. rufipogon and (B) O. nivara, three conserved transactivation domains (domain I, II and III) and one basic leucine zipper (bZIP) domain was predicted. Presence of three protein kinase C phosphorylation site (red pointed serine and threonine), five casein kinase II phosphorylation site (underlined threonine residues) and 11 N-myristoylation sites (indicated in blue colour) were also predicted. The 5-amino acid (AVEHA) deletion in O. rufipogon is indicated with gap. Note that this 5-amino acid sequence is AAEHA in all other genotypes tested except O. nivara and sorghum, which have AVEHA (Fig 1B). (TIF) [file pone.0150763.s006.tif]

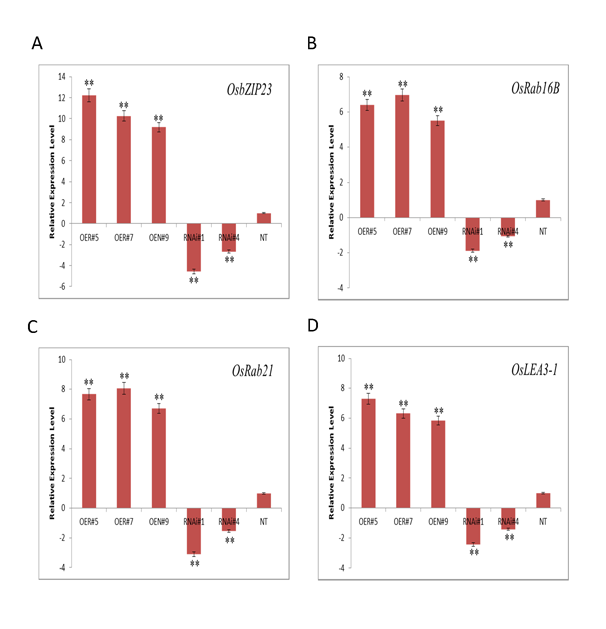

Supplement: S7 Fig — (A) OsbZIP23 (B) OsRab16B (C) OsRab21, and (D) OsLEA3-1 in OsbZIP23 OE lines, RNAi lines and NT plants, where rice polyubiquitin1 (OsUbi1) gene was taken as internal reference. Data bars represent the mean ±SD of triplicate measurement. Statistical analysis by Student’s t-test indicated significant differences (**P<0.01). (TIFF) [file pone.0150763.s007.tiff]

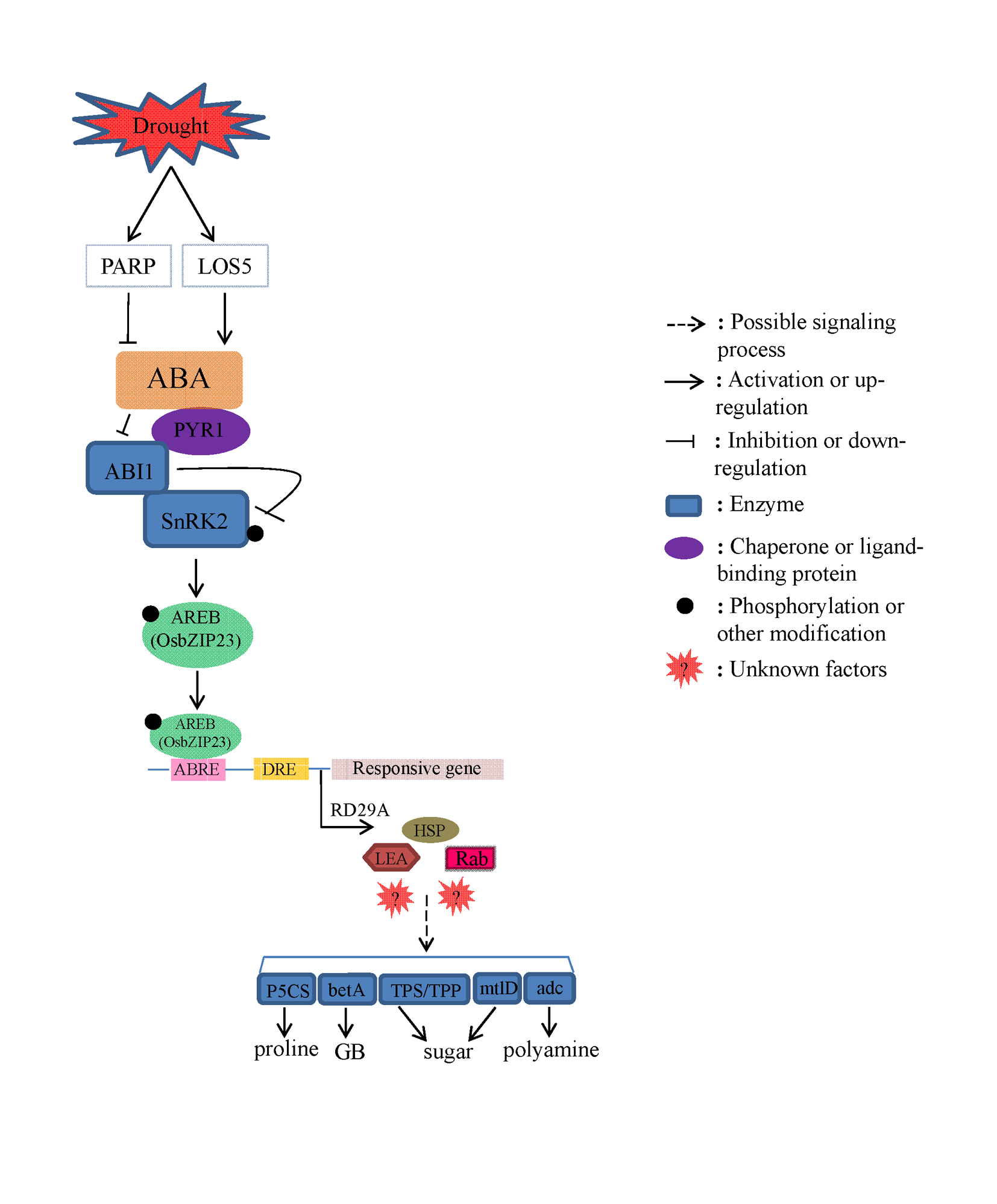

Supplement: S8 Fig — Drought induces accumulation of ABA, which is regulated by PARP and LOS5. ABA is perceived by the PYR1 receptor and induces the expression of SnRK2 kinases by releasing ABI1 phosphatase. This in turn activates the downstream AREB/bZIP-transcription factors. The activated AREBs (through the interaction with ABRE cis-elements) induce the expression of a series of downstream genes to produce different classes of osmoprotectants, which protects the plant by decreasing cellular osmotic potential. Adopted from Yang et al., 2010. (TIFF) [file pone.0150763.s008.tiff]
