# Supplementary material for: Enhanced Gene Expression Rather than Natural Polymorphism in Coding Sequence of the OsbZIP23 Determines Drought Tolerance and Yield Improvement in Rice Genotypes
Source: PLoS One. 2016 Mar 9;11(3):e0150763. doi: 10.1371/journal.pone.0150763 (PMC4784890; doi:10.1371/journal.pone.0150763)
Supplement: S1 Table — (DOCX) [file pone.0150763.s009.docx]

| Purpose Primer Orientation Sequence |
| --- |
| Full length CDS bZ23F Forward ATGGATTTTCCGGGAGGGAGCG  bZ23R Reverse TCACCATGGACCCGTCAGAGTC |
| Full length promoter bZ23PF Forward GCAAGCTTCCTGAACATCTCCTCCACCTTCTT  bZ23PR Reverse TGGGATCCCTCCAAACTCCAACCAACCAATCC |
| Southern hybridization probe GFP-F Forward AGTGGATCCATGGGTAAGGGAGAAGAAC  GFP-R Reverse ACGAGCTCTTATTTGTATAGTTCATCCATGCC  bZ23F Forward AGAAGACCGTCGACGAGGTCT  bZ23R Reverse TCACCATGGACCCGTCAGAGTC |
| Overexpression construct bZIP23F Forward ATTGGATCC ATGGATTTTCCGGGAGGGAGCG  bZIP23R Reverse ATAGGTACCTCACCATGGACCCGTCAGAGTC  RQP-F Forward TCAAGCTTCACATCAGTCTCTGCACAAAGTGC  RQP-R Reverse ATAGGATCCGATCTGCATGCCTCCCCTGAG |
| RNAi construct bZ23Si-F Forward TTGTCGACAGATCACGCTGGAGGAGTT  bZ23Si-F1 Forward TTGGTACCAGATCACGCTGGAGGAGTT  bZ23Si-R Reverse AGGATCCGTCTTCTCTCGACAACCTTCTCGA  LINK-F Forward ATTAGGATCCGCCGAGGCCGCTGCCAAGGAGGCTGCT  LINK-R Reverse ATTAGGATCCGGCCTTGGCAGCGGCCTCCTTGGCAGCA  RQP-F Forward TCAAGCTTCACATCAGTCTCTGCACAAAGTGC  RQP-R Reverse ATTATGTCGACGATCTGCATGCCTCCCCTGAG |
| Real-time PCR bZ23RT-F Forward GGAGCTGAACGATGAACTCCAG  bZ23RT-R Reverse TCGGCTCATTCTCTCTAGAACCTC  OsRab16B-F Forward CAACAACCACCAGCAGCA  OsRab16B-R Reverse GATCTTGTCCATGAATCCC  OsRab21-F Forward AGCAGCAGCATGCCATG  OsRab21-R Reverse TGGTGCCGGTGGTCAT  OsLEA3-1-F Forward TTCCCACCAGGACCAGGCTA  OsLEA3-1-R Reverse GTCGCCTCCTTGGTATCCT  GFP-F Forward GGCCAACACTTGTCACTACT  GFP-R Reverse TTCCTGCACGTATCCCTCA  OsUbi1-F Forward TTGAGGTCGAGTCGTCTGAC  OsUbi1-R Reverse GTGGACTCCTTCTGGATGT |

**Supplementary S1 Table: List of primers used in this study**
